# A new catfish species of *Microcambeva* Costa & Bockmann, 1994 (Siluriformes: Trichomycteridae) from southern Brazil, with a redescription of *M. ribeirae* Costa, Lima & Bizerril, 2004

**DOI:** 10.1111/jfb.70351

**Published:** 2026-03-08

**Authors:** Lucas S. de Medeiros, Igor C. A. Souto‐Santos, Paulo A. Buckup, Juliano Ferrer, Vinicius J. C. Reis, Mario de Pinna, Sergio M. Q. Lima

**Affiliations:** ^1^ Programa de Pós‐Graduação em Sistemática e Evolução, Centro de Biociências Universidade Federal do Rio Grande do Norte Rio Grande do Norte Brazil; ^2^ Laboratório de Ictiologia Sistemática e Evolutiva, Departamento de Botânica e Zoologia, Centro de Biociências Universidade Federal do Rio Grande do Norte Rio Grande do Norte Brazil; ^3^ Centro de Estudos e Monitoramento Ambiental Rio Grande do Norte Brazil; ^4^ Museu Nacional, Universidade Federal do Rio de Janeiro, Museu Nacional, Departamento de Vertebrados, Quinta da Boa Vista Rio de Janeiro Brazil; ^5^ Programa de Pós‐Graduação em Biologia Animal, Departamento de Zoologia, Instituto de Biociências Universidade Federal do Rio Grande do Sul Porto Alegre Brazil; ^6^ Museu de Zoologia da Universidade de São Paulo, Universidade de São Paulo São Paulo Brazil; ^7^ Institut de Systématique, Evolution, Biodiversité, Muséum national d'Histoire naturelle CNRS, Sorbonne Université, EPHE, Université des Antilles CP 30 Paris France; ^8^ Departamento de Biologia Estrutural e Funcional, Instituto de Biociências, Universidade Estadual Paulista “Júlio de Mesquita Filho” Botucatu Brazil

**Keywords:** Atlantic forest, conservation, integrative taxonomy, Microcambevinae, psammophilous fish

## Abstract

Among the few species of *Microcambeva* reported as occurring in more than one hydrographic basin, *M. ribeirae* has been previously listed from both the Ribeira de Iguape and the Guaraqueçaba basins. However, morphological and molecular analyses revealed that the specimens from Guaraqueçaba represent a new species, which is described in this study. Therefore, the distribution of *M*. *ribeirae* is restricted to the Ribeira de Iguape basin, and this taxon is redescribed. The new species is the southernmost record of the genus and is diagnosed among congeners by the T‐shaped dark blotch in the dorsal portion of the head in preserved specimens. It is distinguished from most congeners, except for *M. bendego* and *M. ribeirae*, by the lack of a filamentous first pectoral‐fin ray. Distinguished from the latter species by having only five pre‐caudal vertebrae, the tip of the hyomandibula reaching the vertical through the anterior tip of the preopercle, and the last anal‐fin ray branched. Molecular analysis of the cytochrome c oxidase subunit I (*cox1*) mitochondrial gene including most species of *Microcambeva* (except *M. draco*) supports the close relationship of the new species with *M. ribeirae*. The two species are nonetheless genetically different, with a 2.2% molecular distance. The conservation status and biogeography of the new species and of *M. ribeirae* are discussed.

## 1 INTRODUCTION

The small psammophilous catfishes of the genus *Microcambeva* Costa & Bockmann, 1994 currently comprise eight species distributed from the Jucuruçu basin in Bahia State, in Northeastern Brazil, to Paranaguá bay in Paraná State, in Southern Brazil (Fricke et al., 2025; Medeiros et al., 2021; Sarmento‐Soares et al., 2019). This distribution encompasses four freshwater ecoregions: Northeastern Mata Atlantica, Fluminense, Ribeira de Iguape and Southeastern Mata Atlantica (*sensu* Abell et al., 2008). Species of *Microcambeva* are characterized by their translucent body, reaching a standard length (*L*_s_) of 25 to 50 mm, and are typically found in streams with fast‐flowing water, sandy bottoms, narrow and shallow channels, and moderate riparian vegetation (Costa et al., 2004, 2019; Costa & Bockmann, 1994; Costa & Katz, 2021; Medeiros et al., 2021; Sarmento‐Soares et al., 2019).

Conventional stream sampling methods often neglect the collection of psamophilic fishes. As a result, *Microcambeva* diversity has been largely overlooked, with sparse species descriptions. This scenario has changed in the last decade, a period which saw the description of more than half of the species of the genus (Table 1). Most of these newly described species exhibit similar external morphologies. For instance, *M. mucuriensis* Costa, Katz, Mattos, & Rangel‐Pereira, 2019 and *M. watu* Medeiros, Sarmento‐Soares & Lima, 2021 are morphologically similar to *M. draco* Mattos & Lima, 2010, and *M. bendego* Medeiros, Moreira, de Pinna & Lima., 2020 is similar to *M. ribeirae* Costa, Lima & Bizerril, 2004. Thus, species of *Microcambeva* are usually distinguished by combinations of subtle morphological differences, involving meristic counts, cephalic colour pattern and osteological features, particularly in the mesethmoid and opercular regions (Medeiros et al., 2021).

**TABLE 1:** Chronological list of *Microcambeva* species and their respective river basins and states.

Species	Year	River basin	Ecorregion	State	References
*Microcambeva barbata* Costa & Bockmann, 1994	1994	São João	Northeastern Mata Atlantica	Rio de Janeiro	Costa and Bockmann (1994); Sarmento‐Soares et al. (2019)
*Microcambeva ribeirae* Costa, Lima & Bizerril, 2004	2004	Ribeira de Iguape	Ribeira de Iguape	São Paulo	Costa et al. (2004); Sarmento‐Soares et al. (2019)
*Microcambeva draco* Mattos & Lima 2010	2010	Jucuruçu	Northeastern Mata Atlantica	Bahia	Mattos & Lima (2010); Sarmento‐Soares et al. (2019)
*Microcambeva mucuriensis* Costa, Katz, Mattos & Rangel‐Pereira, 2019	2019	Mucuri	Northeastern Mata Atlantica	Bahia	Costa et al. (2019)
*Microcambeva jucuensis* Costa, Katz, Mattos & Rangel‐Pereira, 2019	2019	Jucu	Northeastern Mata Atlantica	Espirito Santo	Costa et al. (2019)
*Microcambeva bendego* Medeiros, Moreira, de Pinna & Lima, 2020	2020	Guapi‐Macacu	Fluminense	Rio de Janeiro	Medeiros et al. (2020)
*Microcambeva filamentosa* Costa, Vilardo, & Katz, 2020	2020	Ribeira de Iguape	Ribeira de Iguape	São Paulo	Costa, Henschel, & Katz (2020)
*Microcambeva watu* Medeiros, Sarmento‐Soares & Lima, 2021	2021	Doce	Northeastern Mata Atlantica	Minas Gerais and Espirito Santo	Medeiros et al. (2021)

A subgeneric classification of *Microcambeva* has recently been proposed, based on a phylogenetic hypothesis incorporating multigene and morphological data (Costa & Katz, 2021). According to that classification, the genus is divided into three subgenera. The subgenus *Microcambeva* comprises five species: *Microcambeva barbata* Costa & Bockmann, 1994, the type species of the genus, from the Fluminense Ecoregion, *M. draco*, *M. mucuriensis*, *M. jucuensis* Costa, Katz, Mattos & Rangel‐Pereira, 2019 and *M. watu*, from the coastal basins of the Northeastern Mata Atlantica Ecoregion (Costa et al., 2019; Medeiros et al., 2021; Sarmento‐Soares et al., 2019). The monotypic subgenus *Trichocambeva* is represented by *M. filamentosa* Costa, Vilardo, & Katz, 2020, from the Ribeira de Iguape basin and Ecoregion (Costa, Henschel, & Katz, 2020). Lastly, the subgenus *Pterocambeva* includes two species: *M. bendego*, from the Guapi‐Macacu a coastal river basin in the Fluminense Ecoregion, and *M. ribeirae* from the Ribeira de Iguape River basin and ecoregion (Medeiros et al., 2020; Oyakawa et al., 2006; Sarmento‐Soares et al., 2019). However, Costa and Katz (2021) did not include DNA sequences of *M. bendego*, *M. draco* and *M. watu* in their phylogenetic analysis, and the phylogenetic position of these species was established based solely on morphology.

Most species of *Microcambeva* are geographically restricted to a single river basin (Costa et al., 2019; Medeiros et al., 2020, 2021; Sarmento‐Soares et al., 2019), but recent studies reported a form similar to *M. ribeirae* in the Guaraqueçaba basin, located in Paranaguá Bay, in the northern coast of Paraná State, Southern Brazil (Costa & Katz, 2021; Medeiros et al., 2021; Sarmento‐Soares et al., 2019). That record apparently contradicts the presumed basin‐specific distribution of the genus, and prompted doubts on whether it represents an outlying population of *M. ribeirae* or a distinct undescribed species. The present study uses morphological and molecular data of specimens currently identified as *M. ribeirae* from the Ribeira de Iguape and Guaraqueçaba River basins to establish their taxonomic status. Our comparisons also included morphological and molecular data from all species of *Microcambeva*, except for DNA sequences of *M. draco*. We concluded that the lineage from the Guaraqueçaba River basin represents an undescribed species, which is formally described here. A redescription of *M. ribeirae* is also provided, including information on intraspecific variation, colour patterns in life and osteological data, to establish robust diagnostic characters to distinguish it from the new species. As part of the results, a new phylogenetic hypothesis is presented based on molecular data, including sequences of species not previously available, along with information on its biogeography and conservation status.

## 2 MATERIAL AND METHODS

### 2.1 Sampling

Fieldwork expeditions were performed under permit number 12129‐1 issued by the Sistema de Autorização e Informação em Biodiversidade, Instituto Chico Mendes de Conservação da Biodiversidade and historical lots of Museu Nacional (MNRJ), Universidade Federal do Rio de Janeiro and the ichthyological collection of Universidade Federal do Rio Grande do Sul (UFRGS). Our study included specimens and muscle tissue samples from all species of *Microcambeva*, except for *M. draco*. Manipulation, anaesthesia and euthanasia (by immersion in a Eugenol solution) were done according to Brazilian animal welfare laws, guidelines and policies as approved by the Conselho Nacional de Controle e Experimentação Animal (CONCEA, 2013). Whole specimens were fixed in 10% formalin for a week and then transferred to 70% ethanol, or preserved directly in ethanol for DNA extraction, and deposited in the UFRGS and the MNRJ.

### 2.2 Morphological procedures and analyses

Measurements and counts were taken on the left side of specimens using digital callipers under a binocular stereomicroscope, following Costa (1992). Measurements are presented as percentages of standard length (*L*_S_), except for head measurements, which are presented as percentages of head length (*L*_H_). Osteological data and counts of opercular and interopercular odontodes, premaxillary and dentary teeth, procurrent caudal‐fin rays, ribs, branchiostegal rays, and caudal and precaudal vertebrae were obtained from specimens cleared and stained (C&S) according to Taylor and Van Dyke (1985), and from digital radiographs taken at the Departamento de Vertebrados, Museu Nacional, Universidade Federal do Rio de Janeiro, using a Faxitron X‐Ray MX‐20 system.

**FIGURE 1:**
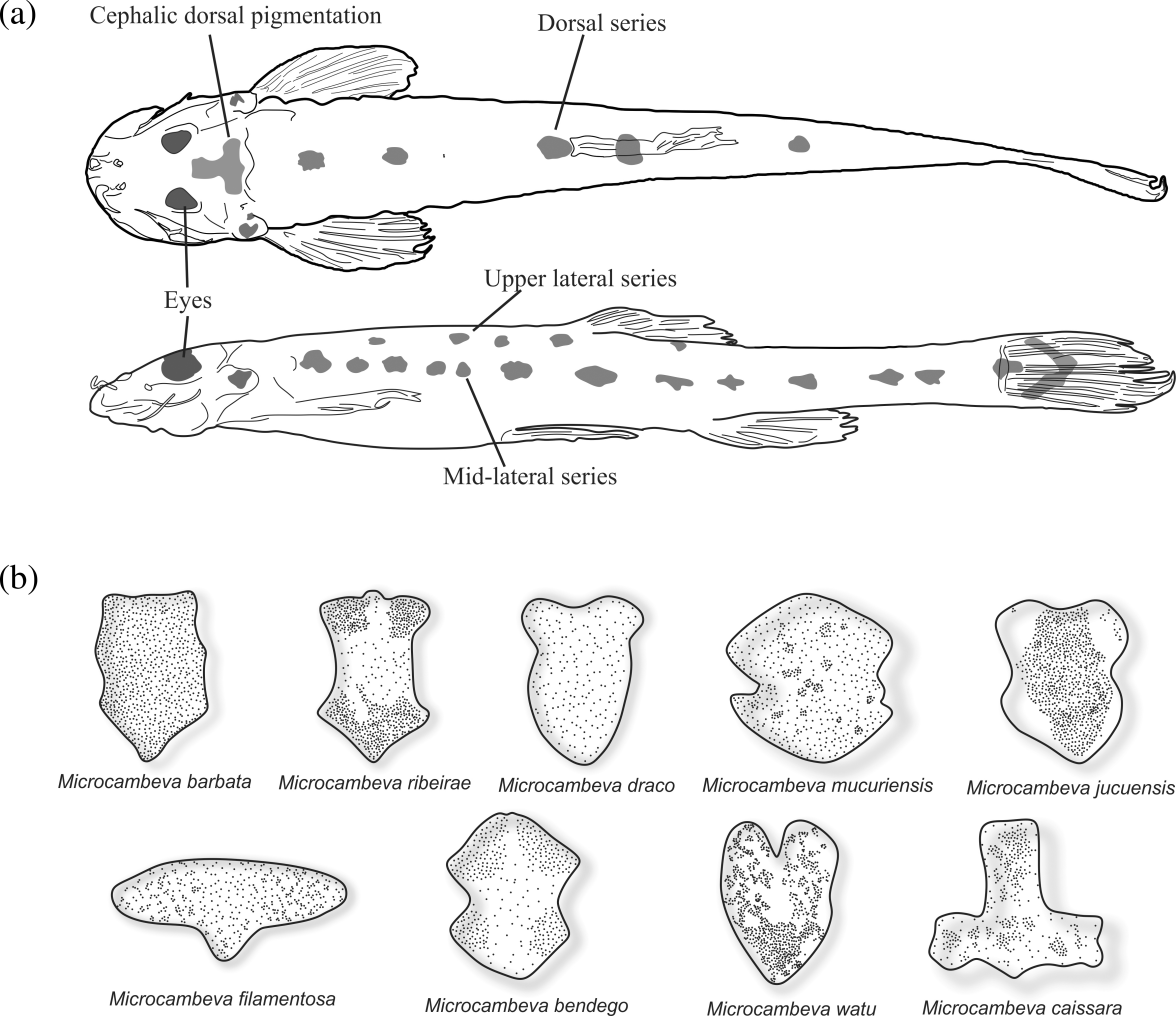
Diagrammatic representation of longitudinal series of dark spots and cephalic dorsal pigmentation patterns in *Microcambeva* species. (a) Schematic depiction of the general distribution of dark spots along the body in dorsal and lateral views. (b) Illustration highlighting dorsal pigmentation pattern on head.

Vertebrae with a complete hemal arch are designated as caudal vertebrae, while those lacking an arch are referred to as pre‐caudal vertebrae. Unbranched fin‐ray counts are indicated in Roman numerals, while branched rays are represented by Arabic numbers. Vertebral counts do not include centra in the Weberian apparatus or the compound caudal centrum. Osteological nomenclature follows de Pinna (1989), except for the nomenclature of bones in the orbital region, which follows de Pinna et al. (2020). Nomenclature of the external anatomical structures of the opercular apparatus follows de Pinna and Dagosta (2022), with additions in de Pinna et al. (2024). Descriptions of colour patterns are based on preserved specimens. The description of the dorsal, upper‐lateral and mid‐lateral longitudinal series of dark chromatophore aggregations are based on comparative examination of their arrangement and distribution across all available preserved specimens. The dorsal series consists of a median row of dark spots aligned longitudinally along the dorsal midline, originating at the occipital region, posterior to the cephalic dorsal pigmentation and extending to the caudal peduncle (Figure 1a). The upper‐lateral series consists of a row of dark spots aligned longitudinally immediately ventral to the dorsal musculature, generally positioned in the epaxial region; this series may exhibit slight variability in spot size and spacing (Figure 1a). The mid‐lateral series comprises a longitudinal row of well‐defined dark spots along the horizontal axis of the flank, from the posterior margin of the opercle to the base of the caudal fin (Figure 1a).

### 2.3 Molecular procedures and analyses

Genomic DNA was extracted from muscle tissue preserved in anhydrous ethanol using the Qiagen Blood and Tissue kit following the manufacturer's instructions, or the salting out method (Miller et al., 1988). DNA quality was verified through standard agarose gel electrophoresis and DNA concentration was measured using a NanoDrop ND‐2000 spectrophotometer. Partial sequences of the mitochondrial gene cytochrome oxidase subunit I (*cox1*) were amplified via the polymerase chain reaction (PCR) using FishF1 (5′ TCA ACC AAC CAC AAA GAC ATT GGC AC 3′) and FishR1 (5′ TAG ACT TCT GGG TGG CCA AAG AAT CA 3′) primers developed by Ward et al. (2005). The amplified products were verified using 2% agarose gel electrophoresis. The PCR products were purified by using Exo‐SAP (Bell, 2008) or the QIAquick Purification Kit (Qiagen) at Macrogen. Each PCR product was sequenced bidirectionally on an ABI3730xl (Applied Biosystems) automated sequencer at the Fundação Oswaldo Cruz (FIOCRUZ) or on an ABI PRISM®3500 (Applied Biosystems) at Macrogen.

Our samples included 14 terminal taxa from three Trichomycteridae subfamilies and additional sequences from GenBank (Table 2). Ingroup taxa comprise eight of nine species of *Microcambeva*. DNA sequences of *M. barbata*, *M. filamentosa, M. jucuensis*, *M. mucuriensis* and *M. ribeirae* were obtained from GenBank (Table 2), while sequences of *M. bendego*, *M. watu*, and the new species were obtained in this study. Outgroup taxa include *Trichogenes longipinnis* Britski & Ortega, 1983 (Trichogeninae), *Trichomycterus nigricans* Valenciennes, 1832 (Trichomycterinae), *Cambeva davisi* (Eigenmann, 1918) (Trichomycterinae), *Listrura tetraradiata* Landim & Costa, 2002, *L. picinguabae* Villa‐Verde & Costa, 2006, and *L. costai* Villa‐Verde, Lazzarotto & Lima, 2012 (Microcambevinae). Accession codes of sequences are provided in Table 2.

**TABLE 2:** GenBank accession codes of cytochrome c oxidase subunit 1 *(cox1)* sequences used to generate the species tree and genetic distances among species of *Microcambeva*.

Species	Voucher	GenBank accession	Source
Trichogeninae			
*Trichogenes longipinnis*	UFRJ 10295	MK123682.1	Katz et al. (2018)
Trichomycterinae			
*Trichomycterus nigricans*	UFRJ 10989	MN385796.1	Costa, Henschel, & Katz (2020)
*Cambeva davisi*	LBP 7130	KY857988.1	Ochoa et al. (2017)
Microcambevinae			
*Microcambeva barbata*	UFRJ 12185 MNRJ47108 MNRJ47108	MN385804.1 PP213822 PV980089	Costa, Vilardo, & Katz (2020) This study This study
*Microcambeva jucuensis*	UFRJ 11011	MN385805.1	Costa, Vilardo, & Katz (2020)
*Microcambeva mucuriensis*	UFRJ 11028	MN385806.1	Costa, Vilardo, & Katz (2020)
*Microcambeva watu*	MBML 4383	PV945362	This study
*Microcambeva filamentosa*	UFRJ 12180	MN385808.1	Costa, Henschel, & Katz (2020)
*Microcambeva ribeirae*	UFRJ 12179	MN385807.1	Costa, Vilardo, & Katz (2020)
*Microcambeva caissara*	MNRJ 32443 UFRGS 24759 UFRGS 24759 MNRJ 53822	PP213825 PV980076 PV980077 PP213824	This study This study This study This study
*Microcambeva bendego*	MNRJ 48616	PP213823	This study
*Listrura costai*	MNRJ39620	HM245412.1	Villa‐Verde et al. (2013)
*Listrura tetraradiata*	MNRJ 39068	JQ231083.1	Villa‐Verde et al. (2013)
*Listrura picinguabae*	LBP 3864	KY857951.1	Ochoa et al. (2017)

Sequences were manually edited using Geneious v.6 software (http://www.geneious.com) to fine‐tune base calls and ensure codon alignment, and aligned using the ClustalW algorithm (Chenna et al., 2003). The original sequences have 655 base pairs, but are trimmed to 521 nucleotides in the alignment to eliminate missing data regions. Gene divergence between sequences was calculated in MEGA X (Kumar et al., 2018), under the Kimura 2‐parameter (K2P) model (Kimura, 1980). Phylogenetic trees were inferred by the Bayesian Inference (BI) analyses using the BEAST 2.5 software (Bouckaert et al., 2019), with the HKY + I + G nucleotide substitution model, which was selected based on the Akaike criterion using the jModeltest 2 software (Darriba et al., 2012). The analyses utilized a strict clock lognormal model, along with default parameter values. A Markov Chain Monte Carlo was executed for 10,000,000 generations, with samples taken at every 1000 runs. To assess the effective sample size, the Tracer 1.7.2 software was employed, and values greater than 200 were regarded as acceptable (Rambaut et al., 2018). The initial 1000,000 trees were discarded, and a consensus tree, as well as posterior probabilities, were obtained using the TreeAnnotator v.1.10.4 software. The consensus tree was edited using TreeViewer v2.2.0 software (Bianchini & Sánchez‐Baracaldo, 2024). The consensus tree was then used to create a geophylogeny of lineages with respect to geographic distribution using the GenGIS v. 2.5.3 software (Parks et al., 2013).

## 3 RESULTS

### 3.1 *Microcambeva caissara,* new species, Medeiros, Souto‐Santos, Buckup, Ferrer, Reis, de Pinna & Lima

urn:lsid:zoobank.org:act:7FE1D163‐B836‐4702‐B0AB‐54188924D9C7.

**FIGURE 2:**
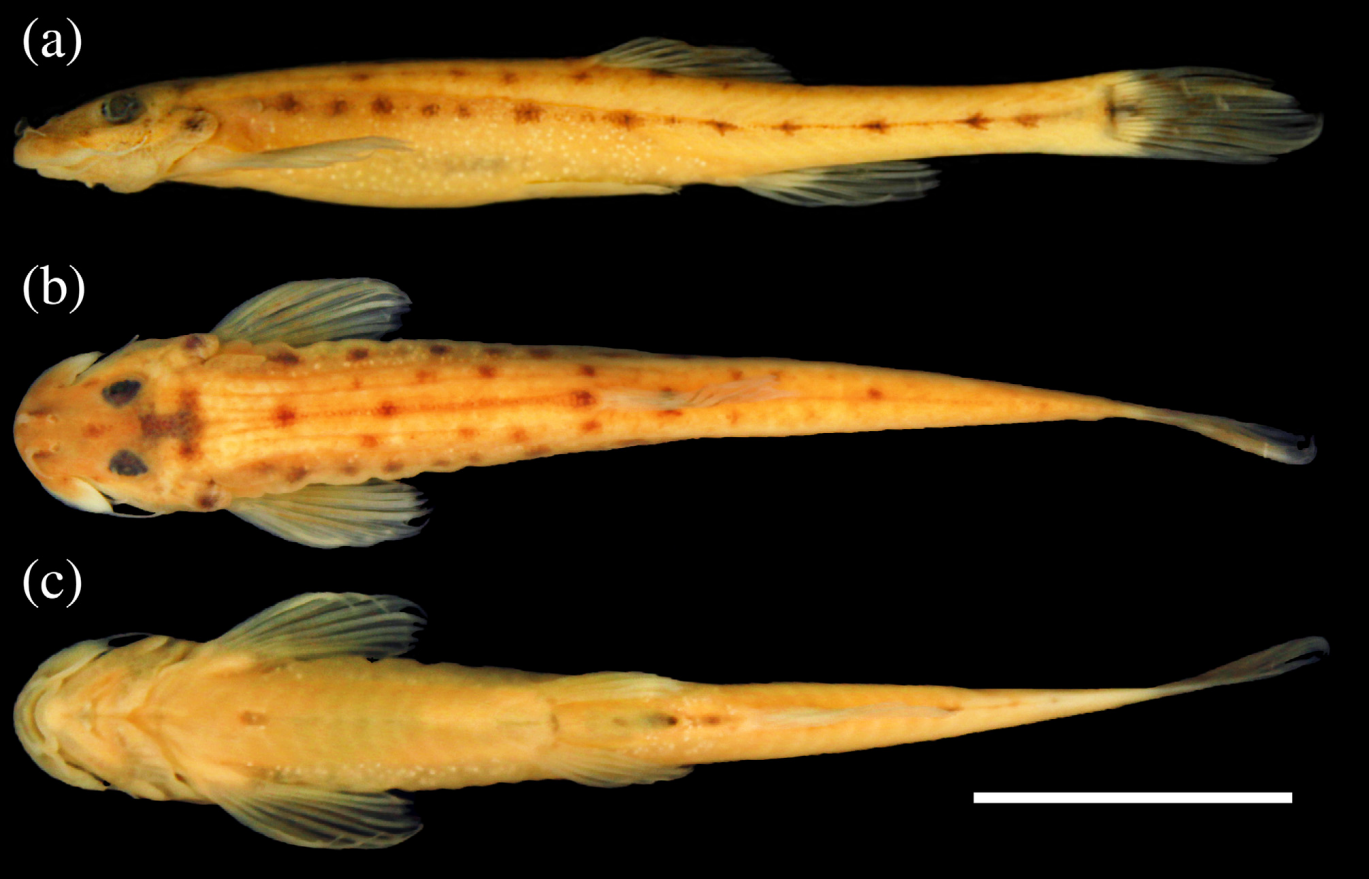
*Microcambeva caissara*, holotype, UFRGS 29330, 37.3 mm *Ls*, affluent of Guaraqueçaba River at road PR‐405, Guaraqueçaba River basin, Guaraqueçaba, Paraná, Brazil: (a) left lateral view, (b) dorsal view, (c) ventral view. Scale bar: 10 mm.

**FIGURE 3:**
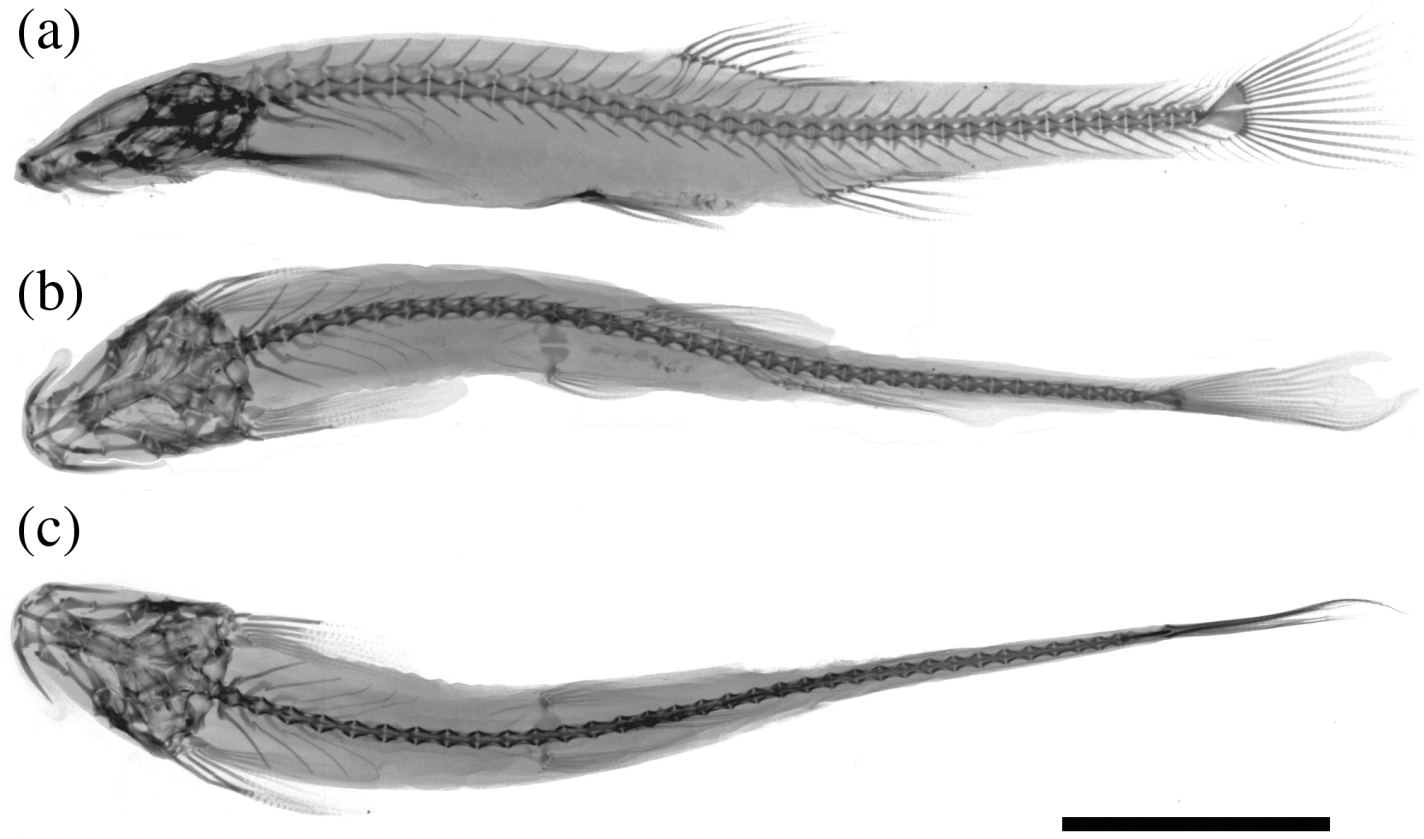
Radiography of *Microcambeva caissara*, MNRJ 53822, 41.7 mm *Ls*, paratype: (a) left lateral view, (b) dorsal view, (c) ventral view. Scale bar: 10 mm.

**FIGURE 4:**
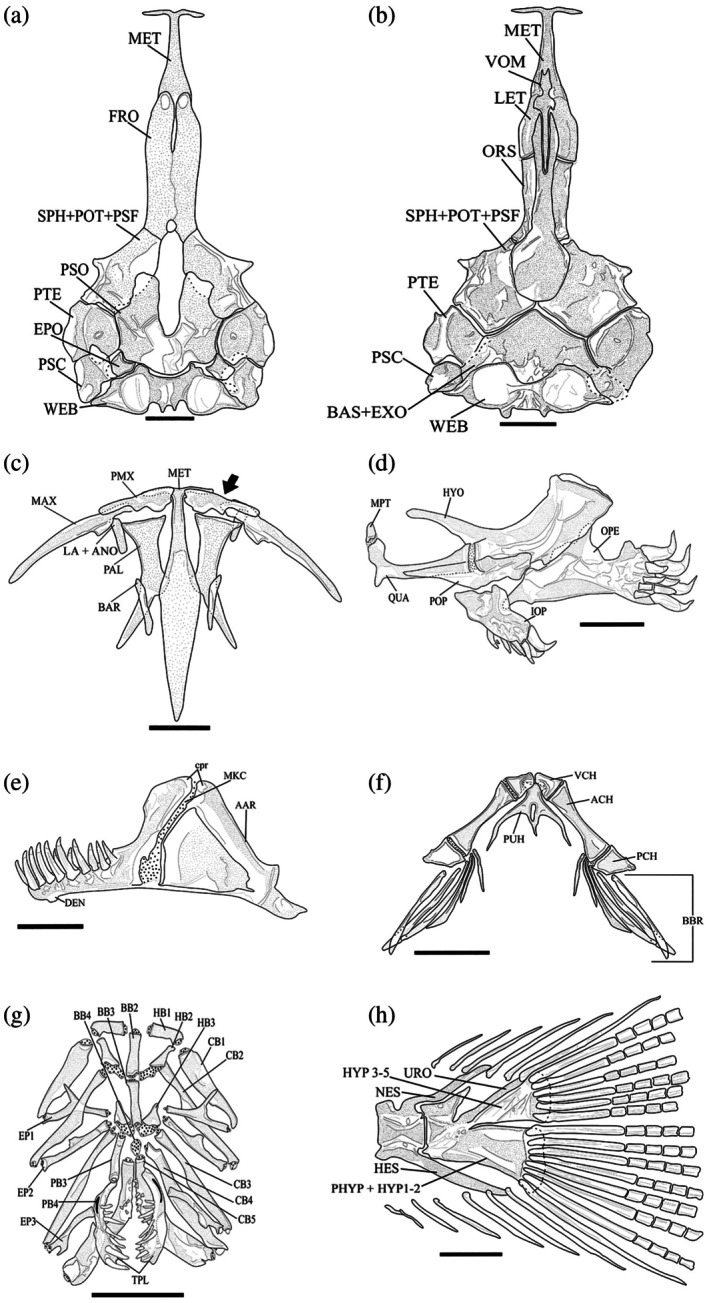
Skeleton of *Microcambeva caissara*, MNRJ 32443, 30.4 mm *L*_S_, paratype. Skull and Weberian apparatus in (a) dorsal and (b) ventral view. (c) Dorsal view of upper jaw and associated structures. (d) Suspensorium and opercular apparatus, left side, lateral view, (e) lower jaw, left side, external view, (f) hyoid arch, ventral view, (g) branchial skeleton, ventral view, (h) caudal skeleton, left side, ventral view. AAR, anguloarticular‐retroarticular; ACH, anterior ceratohyal; BAR, barbular; BAS + EXO, basioccipital + exoccipital; BB2‐4, basibranchials 2–4; BRR, branchiostegal rays; CB1‐5, ceratobranchials 1–5; cpr, coronoid process; DEN, dentary; EPO, epioccipital; EP1‐4, epibranchials 1–4; FRO, frontal; HB1‐3, hypobranchials 1–3; HES, haemal spine; HYO, hyomandibula; HYP 1–2 + PH, hypurals 1–2 + parahypural fused; HYP 3–5, hypurals 3–5 fused; ICH, interceratohyal cartilage; IOP, interopercle; LA + ANT: Lacrimal+antorbital LET, lateral ethmoid; MAX, maxilla; MET, mesethmoid; MKC, meckel's cartilage; MPT, metapterygoid; NES, neural spine; OPE, opercle; ORB, orbitsphenoid; PAL, palatine; PAS, parasphenoid; PB3‐4, pharyngobranchials 3–4; PCH, posterior ceratohyal; PHYP, parahypural; PMX, premaxilla; POP, preopercle; PSC, Posttemporo‐supracleithrum; PSO, Parieto‐supraoccipital; PTE, Pterotic; PUH, parurohyal; QUA, quadrate; SPH + POT + PSF: Sphenotic + Prootic + Pterosphenoid complex; TPL, toothplate; URO, uroneural spine; VHH, ventral hypohyal; VOM, vomer; WEB, capsule of weberian apparatus. Black arrows indicate the lateral process of premaxilla short, approximately at same length of bone without lateral process, and rounded (b). Scale bar: 1 mm, excepted for, lower jaw which is 0.5 mm.

**FIGURE 5:**
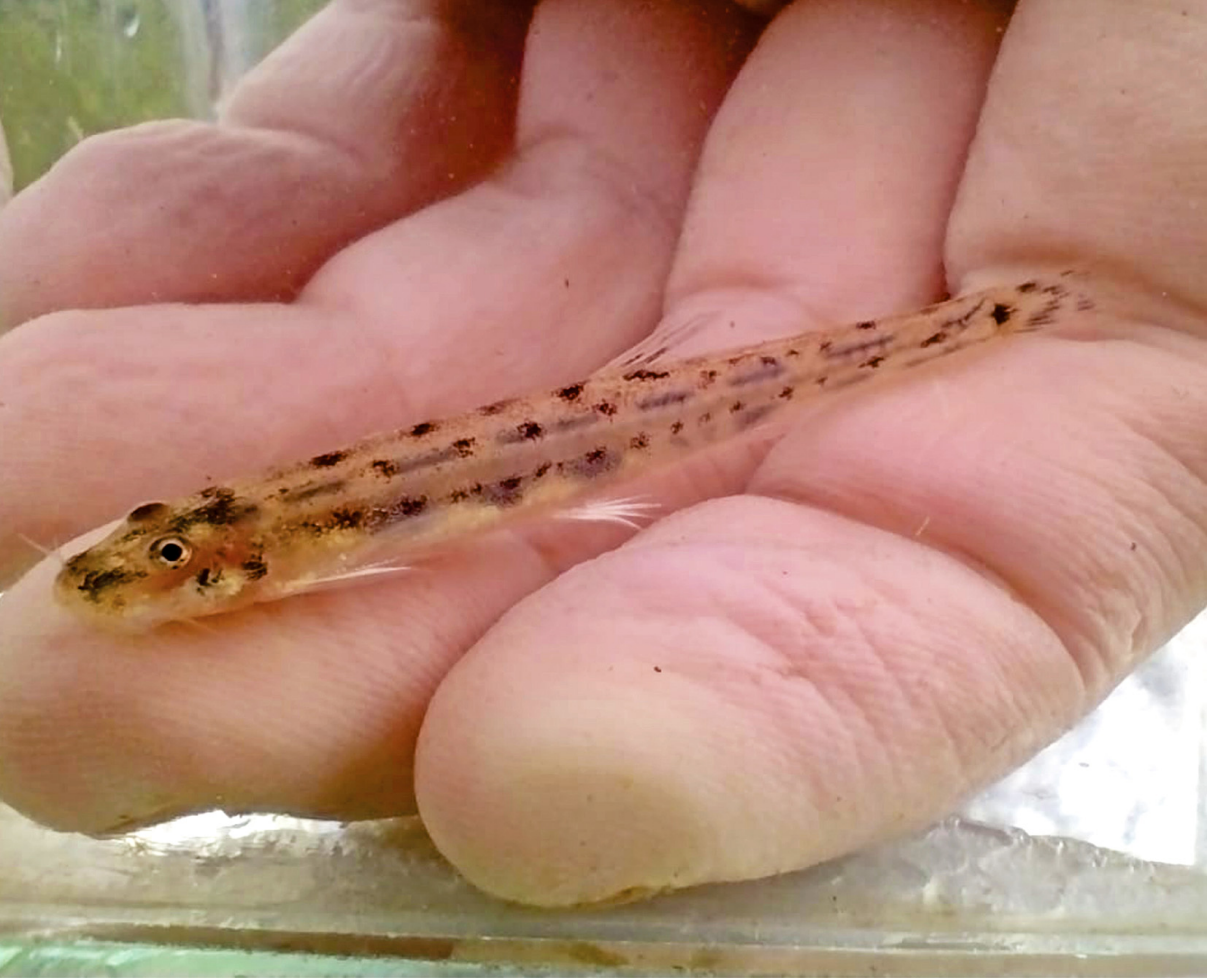
Live specimen of *Microcambeva caissara*, MNRJ 53822, 41.7 mm *L*_S_, from Guaraqueçaba River basin, Paraná State, southern Brazil.

Figures 2, 3, 4, 5 and Table 3.

**TABLE 3:** Morphometrics data of the holotype of *Microcambeva caissara* (UFRGS 29330), paratypes (UFRGS 24759; MNRJ 32443; MNRJ 53822), *M. ribeirae* (holotype MZUSP 84301 and additional material, including paratypes, see the section of Examined Material).

	*Microcambeva caissara*				*Microcambeva ribeirae*			
	Holotype	Range	Mean	*n*	Holotype	Range	Mean	*n*
Standard length	37.3	14.6–41.7	26.56	16	47.8	25.9–47.8	36.5	43
	Percentage of *L*_s_							
Body depth	12.5	8.9–29.0	12.0	16	11.7	8.7–15.2	11.3	43
Caudal peduncle depth	7.0	5.5–23.7	7.7	16	7.5	5.2–8.9	6.9	43
Body width	9.7	4.2–9.7	6.7	16	9.2	5.7–12.3	7.9	43
Caudal peduncle width	4.0	2.6–5.3	4.1	16	4.3	2.4–7.5	4.6	43
Caudal peduncle length	15.5	13.7–30.2	16.7	16	13.8	10.9–29.0	16.6	43
Dorsal‐fin base length	8.7	4.5–11.0	8.0	16	10.1	7.2–12.5	10.1	43
Anal‐fin base length	7.1	4.8–11.1	6.7	16	6.4	5.0–11.1	7.4	43
Pelvic‐fin length	13.0	4.7–18.0	12.9	16	14.2	12.8–17.5	14.6	43
Distance between pelvic‐fin bases	3.8	1.6–13.5	4.2	16	3.1	1.6–7.1	3.7	43
Pectoral‐fin length	18.3	14.4–23.0	19.7	16	6.9	7.0–24.5	19.7	43
Pre‐dorsal length	52.3	47.4–58.3	53.2	16	52.2	46.3–58.5	52.5	43
Pre‐pelvic length	47.5	39.7–70.5	57.5	16	41.8	41.0–52.8	45.8	43
Head length (*L*_H_)	16.1	16.1–23.0	20.5	16	18.7	16.8–21.7	19.5	43
	Percentage of *L*_H_							
Head depth	56.2	30.7–56.2	44.3	16	46.9	31.4–60.2	46.9	43
Head width	84.9	67.5–92.0	82.0	16	78.8	67.0–97.6	82.4	43
Interorbital width	19.0	11.6–31.1	17.1	16	20.3	11.4–20.9	15.1	43
Preorbital length	56.2	37.5–56.5	41.8	16	43.5	32.6–50.1	41.5	43
Eye diameter	22.0	17.7–28.5	22.0	16	17.7	15.0–29.6	19.3	43
Mouth width	24.4	13.8–28.6	23.6	16	34.0	13.4–34.0	25.2	43
Inter‐nares width	15.8	5.8–15.9	10.1	16	13.5	5.6–28.6	11.1	43

Abbreviation: n, number of specimens analyzed.

*Microcambeva ribeirae* (not Costa et al., 2004). Sarmento‐Soares et al., 2019: 585–586 (geographic record of a specimen, misidentified as *M. ribeirae*); Medeiros et al., 2020: 121 (listed a specimen, misidentification). Medeiros et al., 2021: 12 (listed a specimen, misidentification). Costa & Katz, 2021: 319 (*in partim*: geographic distribution).

#### 3.1.1 Holotype

UFRGS 29330, 37.3 mm *L*_S_, Brazil, Paraná State, Municipality of Guaraqueçaba, Cedro River, tributary of left margin of Guaraqueçaba River basin, at road PR‐405, (25°09′28.4′′S, 48°13′42.8′′W); col. L. M. Donin, N. Pio, P. M. Ito, T. P. Carvalho; 12 December, 2017.

#### 3.1.2 Paratypes

All specimens from Brazil, Paraná State, Municipality of Guaraqueçaba, Guaraqueçaba River basin: UFRGS 24759, 6 alc (TEC 8317) and 1 C&S, 21.2–38.5 mm *L*_S_, collected with holotype. MNRJ 32443, 9 alc. (MNTI 1117) and 2 C&S, 29.8–30.5 mm *L*_S_, Cedro River, tributary to the left margin of the Guaraqueçaba River, between Guaraqueçaba and Bataba, (25°09′28″S, 48°13′41″W); col. P. A. Buckup, M. R. Britto, L. Villa‐Verde, R. Bartolette, L. F. S. Ingenito, L. Duboc, J. M. Santos, S. P. Castro; 9 April 2008. MNRJ 53822, 1 alc (MNTI 15431), 41.7 mm *L*_S_, Morato River, near bridge of PR‐405 road, (25°12′48″S, 48°17′53″W); col. P. A. Buckup, I. C. A. Souto‐Santos, J. E. Mejía, G.A. Ferraro; 4 February 2022.

#### 3.1.3 Diagnosis

*Microcambeva caissara* differs from all congeners by the T‐shape dark blotch in the posterior dorsal portion of the head, which is conspicuous in the dorsal view of preserved specimens. This effect is a visual combination of a median portion formed by brain pigment seen by transparency and two integumentary spots on each side of the posterior end of the dark brain pigmented area (Figure 1b, vs. cephalic hourglass‐shaped in *M. bendego* and *M. ribeirae*, ellipsoid in *M. filamentosa*, triangular with constriction near anterior portion in *M. draco*, hexagonal with median constriction in *M. mucuriensis*, pentagonal with median constriction in *M. jucuensis*, diamond‐shaped in *M. barbata* and heart‐shaped in *M. watu*). The new species is further distinguished from congeners, except *M. ribeirae* and *M. bendego*, by lacking a filamentous extension of first pectoral‐fin ray (vs. ray extending as filament). It is distinguished from *M. ribeirae* and *M. bendego* by having the last anal‐fin ray branched (vs. unbranched) and five pre‐caudal vertebrae (vs. six). It differs further from *M. bendego* by elongation of the anterior process of the opercle (vs. short process). It is further distinguished from *M. ribeirae* by tip of maxillary barbel reaching to anterior portion of the interopercular odontodophore (vs. maxillary barbel tip extending beyond posterior margin of interopercular odontodophore); by lateral process of premaxilla short, approximately as long as the remainder of the bone, and round (vs. lateral process approximately twice as long as the remainder of the bone, and pointed).

#### 3.1.4 Description

Morphometric data of holotype and paratypes in Table 3. Body elongated, head wider than trunk in dorsal view (Figure 2a,c). Cross‐section of body cylindrical posterior to head, progressively compressed at pelvic‐fin insertion, tapering toward base of caudal fin. In lateral view, lowest body depth posterior to head, greatest approximately at dorsal‐fin origin. Dorsal body profile slightly convex from tip of snout to dorsal‐fin origin; straight along caudal peduncle, slightly concave at dorsal procurrent rays. Ventral body profile convex from gular region to pelvic‐fin origin; straight between urogenital opening and caudal peduncle; slightly concave at ventral at caudal peduncle.

Head depressed, triangular in dorsal view, longer than wide, less deep than body (Figure 2b). Mouth subterminal, wide; upper jaw longer than lower (Figure 2c). Barbels similar to each other in general aspect, their internal cores visible by transparency. Anterior nostril small, round, located closer to upper lip than to anterior margin of eye, surrounded by small tube of integument, continuous posterolaterally with nasal barbel. Posterior nostril round, larger than anterior one, situated slightly closer to eye than to anterior nostril, surrounded by low rim of integument. Maxillary barbel originating laterally at upper lip, wide at base, progressively tapering to fine distal tip reaching posterior portion of interopercular odontodophore (*n* = 16, including holotype). Rictal barbel originating laterally at lower lip, its tip reaching anterior portion of interopercular odontodophore (*n* = 16, including holotype). Pair of finger‐like barbels (sensu Medeiros et al., 2020) approximately half size of eye, set closely to branchial isthmus. Nasal barbel originating at latero‐median portion of anterior nostril, wide at base, tapering distally, reaching posterior region of posterior nostril (*n* = 16, including holotype). Eye round, wide, covered by thin, transparent skin, located dorsolaterally on head. Interorbital nearly twice longitudinal diameter of eye. Branchiostegal membranes narrowly united to isthmus. Five pleural ribs. Free vertebrae, 32 (27 caudal, 5 pre‐caudal; Figure 3).

Pectoral‐fin rays seven (i + 6) (*n* = 10, including holotype) or eight (i + 7) (*n* = 6); first ray unbranched, not prolonged as filament, approximately 40% shorter than other rays; remaining rays branched, progressively longer than membrane; tips of rays forming continuous line along fin margin (Figure 2b). Axillary gland located dorsoposteriorly of pectoral‐fin insertion. Dorsal‐fin origin at vertical through 13th vertebrae, with nine rays (ii + 6 + i) (*n* = 14, including holotype). Pelvic‐fin origin at vertical through 10th vertebrae, well separated from each other at base, their tips extending beyond urogenital papilla; five pelvic‐fin rays (i + 4) (*n* = 16, including holotype). Anal fin larger than dorsal fin; its origin at vertical through 18th vertebrae. Seven anal‐fin rays (ii + 4 + i) (*n* = 16, including holotype). Caudal fin truncated, with 13 rays (i + 11 + i) (*n* = 16, including holotype), six principal rays (i + 5) on dorsal and seven (6 + i) on ventral hypural plate. Dorsal and ventral procurrent caudal‐fin rays six; ventral rays longer than dorsal ones.

Mesethmoid elongated; its main axis and anterior margin mostly straight; posterior portion pointed, overlapped posterodorsally by frontal; mesethmoid cornua short, straight, with pointed tips (Figure 4a). Frontal slender, short. Cranial fontanel round anteriorly and posteriorly. Sphenotic, pterosphenoid and prootic fused into continuous trapezoid bone. Vomer long, arrow‐shaped; anterior portion small, with lateral constriction, minute lateral projections anteriorly to constriction, with tips posteriorly directed; vomer broadest at lateral projections; posterior shaft long, pointed (Figure 4b). Lateral ethmoid rectangular, without lateral projections. Parasphenoid narrow anteriorly, wide posteriorly; anterior extremity bifurcated around vomer; posterior extremity oval. Basioccipital fused with exoccipital and Weberian complex posteriorly.

Premaxilla triangular, with elongated toothless lateral process ca. 40% of total premaxillary length. Conspicuous protuberance on dorsal surface of posteromedial portion of premaxilla near palatine cartilage (Figure 4c). Premaxillary teeth 19 on each side, conical, arranged in two rows. Maxilla, narrow, elongate, with pointed tips; 50% longer than premaxilla (including lateral process). Dentary teeth 30, small, conical, arranged in two rows of 15 each (Figure 4e). Palatine long, ending posterolaterally in elongated pointed process, nearly as long as antero‐medial portion of palatine. Process straight, its lateral margin with pronounced concavity. Lacrimal‐antorbital cylindrical, elongated, approximately 65% as long as barbular. Barbular cylindrical, elongated.

Metapterygoid elliptic (Figure 4d). Quadrate elongate, with small pointed process extending anterodorsally from anterior tip; its dorsal margin gently concave, horizontally directed. Hyomandibula with narrow, pointed, elongated anterior process curved anteriorly at anterior portion, its tip at vertical through anterior tip of preopercle. Preopercle straight, elongate, tapering anteriorly, approximately 20% longer than quadrate; posterior edge roundish. Interopercle with eight conical odontodes obliquely arranged in two irregular rows. Opercle slender, with nine conical odontodes arranged obliquely in two or three irregular rows, pronounced dorsomedial concavity, large ascending process anteriorly.

Parurohyal with short posterior process, shorter than lateral wings (Figure 4f); its central foramen small, ellipsoid. Ventral hypohyal trapezoid, with deep fossa on ventral surface for articulation with parurohyal condyle. Anterior ceratohyal long, rod‐like, constricted at middle. Posterior ceratohyal sub‐triangular. Branchiostegal rays 6. Basibranchial 1 absent. Basibranchial 2–3 rod‐shaped (Figure 4g). Basibranchial 4 fully cartilaginous, roundish, depressed. Hypobranchial 1, rod‐shaped, with distal portion anteriorly‐directed; hypobranchials 2 and 3 conical. Ceratobranchials 1 to 4 cylindrical, gently concave distally; ceratobranchial 2 gently concave at proximal portion; ceratobranchial 5 rectangular, with small ventroposterior process; ceratobranchials 1 and 2 with small triangular expansion at ventromedial region; ceratobranchial 5 with six conical teeth irregularly arranged along middle portion. Epibranchial 1 Y‐shaped; epibranchial 2 rod‐shaped, with lateral expansion, anteriorly‐directed, along middle of dorsal portion; epibranchial 3, with distal laminar expansion at antero‐ventral portion, posteriorly directed; epibranchial 5 rectangular. Pharyngobranchials 1 and 2 absent; pharyngobranchial 3 rod‐like; pharyngobranchial 4 cartilaginous, inserted at laterodorsal margin of lower pharyngeal tooth plate. Pharyngeal tooth plate with seven to nine conical teeth in single row.

Hypural complex with two plates. Ventral plate rectangular, formed by fusion of parhypural plus hypurals 1 and 2; dorsal plate formed by fusion of hypurals 3 to 5 (Figure 4h). Uroneural elongate, as long as adjacent neural spine, not fused to dorsal plate.

#### 3.1.5 Colour in ethanol

Dorsal, lateral and ventral surfaces of body yellowish pale (Figure 2). Dorsal series with five spots irregularly spaced; first spot posterior to occipital region; third spot at dorsal‐fin insertion; fourth spot posterior to dorsal‐fin insertion; fifth spot located on middle portion of caudal peduncle. Upper‐lateral series with five or six spots. Mid‐lateral series with 13 unevenly spaced spots; first spot dorsal to base of pectoral fin, last spot largest, oval, covering hypural plate and central region of caudal‐fin base. Posterior portion of dorsal surface of head with large dark T‐shaped mark, extending from slightly anteriorly to line through posterior margin of eyes to anterior limit of epaxial musculature, formed by combination of brain pigment (corresponding to the stalk of the ‘T’) and paired integumentary dark spots (corresponding to the transversal bar of the ‘T’). Eyes and iris brownish. Slender dark field extending between anterior and posterior nostrils. Remainder of dorsal surface of head with light uniform covering of brownish chromatophores. Intensely dark concentration of chromatophores on opercular odontodophore. Ventral surface of head white. Maxillary and nasal barbels with small dark chromatophores dispersed at base. Base of barbels yellowish and white along thinner portion. Pale‐brown, V‐shaped bar on caudal‐fin base, with angle of ‘V’ directed posteriorly.

#### 3.1.6 Colour in life

Dorsum and trunk translucent. Ventral surface of body pale yellowish. Series of large dark spots along body (Figure 5). Small melanophores distributed irregularly along body. Mid‐lateral and upper lateral series of spots with appearance similar to that described for preserved specimens. Dorsal series with four spots irregularly spaced between occipital region and dorsal‐fin origin; first spot small posterior to occipital cephalic blotch; fourth spot anterior to dorsal‐fin origin; fifth spot posterior to dorsal‐fin base. Two spots at caudal peduncle, one at posterior portion of peduncle, another at caudal‐fin base. Head with large dark blotch extending over middle of posterior surface of neurocranium. Eyes black, iris yellowish. Ventral surface of head uniform yellowish. Slender brownish blotch extending laterally between anterior and posterior nostrils. Barbels and fins hyaline. Pale brown, V‐shaped bar on caudal‐fin base, with V‐shaped angle of ‘V’ directed posteriorly.

#### 3.1.7 Etymology

The term ‘*caiçara*’ (‘*caá‐içara*’) originates from the Tupi‐Guarani indigenous language and refers to the wood stakes placed around villages or to a fish corral (Adams, 2000; Sampaio, 1987). Over time the name was applied to other concepts, for example huts built on to shelter small canoes or a fishing instrument, and as gentilic to those born in the Cananeía region, in the southern coastal of São Paulo State. Nowadays, Caiçara is commonly used to refer to all natives and communities on the coast of the States of Paraná, São Paulo and Rio de Janeiro (Diegues, 1983).

#### 3.1.8 Geographic distribution and ecology

**FIGURE 6:**
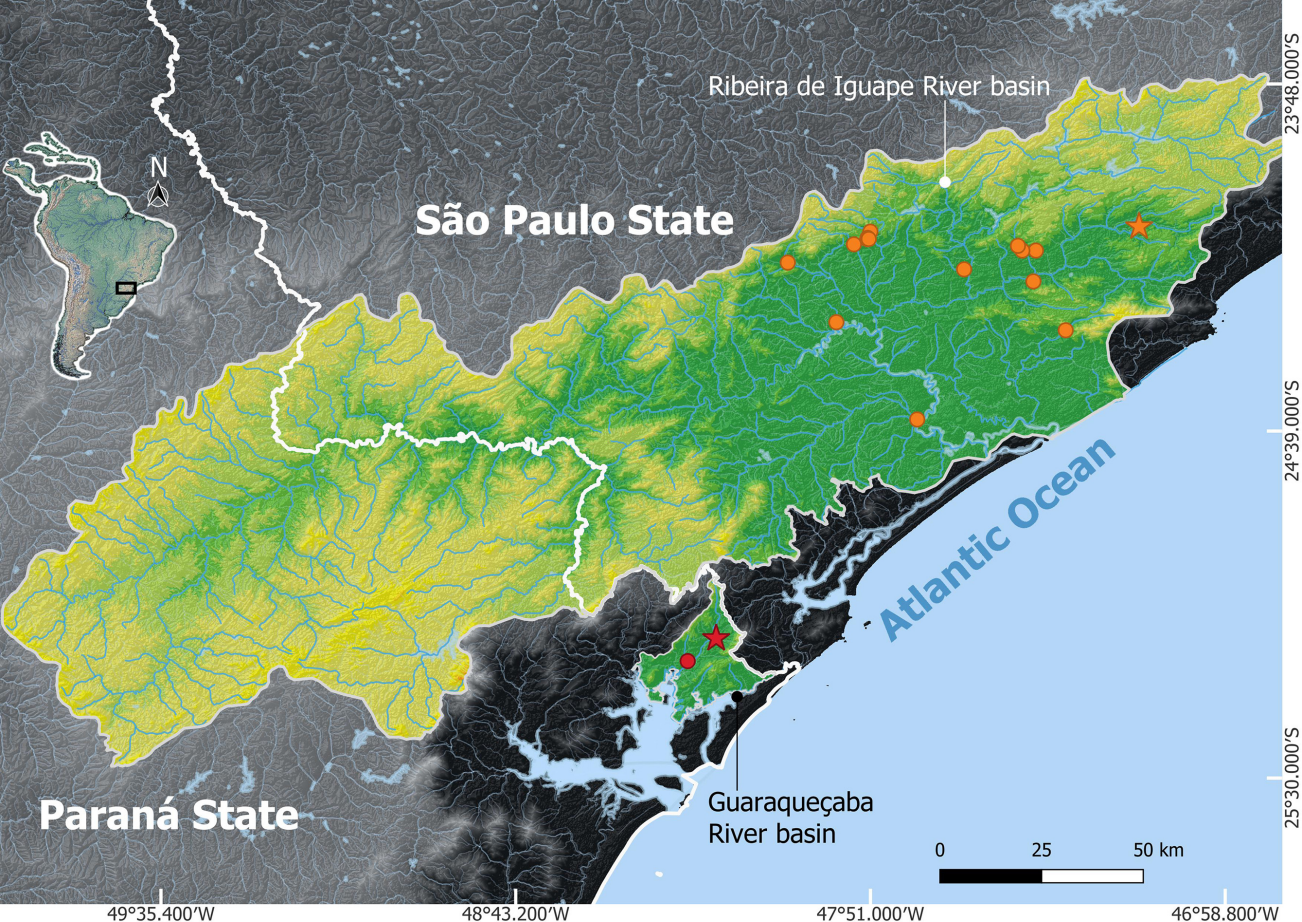
Geographic distribution of *Microcambeva caissara*, in Guaraqueçaba River basin, Paraná State, and *M. ribeirae* in Ribeira de Iguape River basin, São Paulo State. Red star ‐holotype and paratype locality of *M. caissara*; red circle ‐ paratype locality; orange star ‐ holotype and paratype localities of *M. ribeirae*; orange circles ‐ additional records of *M. ribeirae*; white line, state limits; blue lines, hydrography.

**FIGURE 7:**
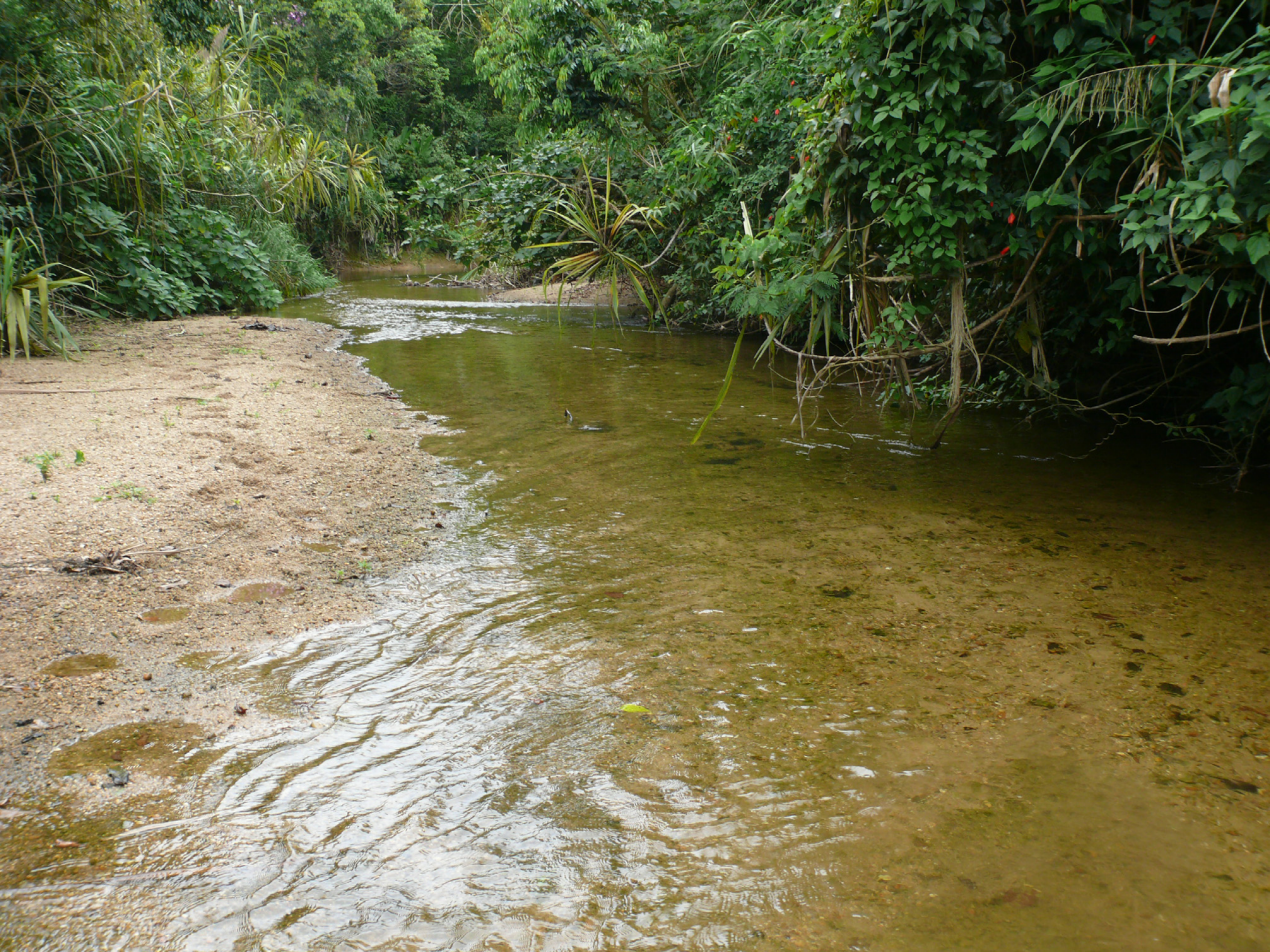
Type locality of *Microcambeva caissara*, Brazil, Paraná State, Municipality of Guaraqueçaba, Cedro River, tributary of the left margin of Guaraqueçaba River, at road PR‐405 (photo by Tiago P. Carvalho).

*Microcambeva caissara* is only known from the Morato and Cedro rivers, tributaries of the middle portion of the Guaraqueçaba River Basin, at the Municipality of Guaraqueçaba in the Paraná State, Brazil, in the southeastern Mata Atlantica ecoregion (Figure 6). According to the Köppen scale, the region has a humid subtropical climate classified as Cfa (Peel et al., 2007). The new species inhabits small, shallow, moderate‐flowing streams surrounded by dense forest. The holotype and some paratypes (UFRGS 24579) were collected during the day in a clear water stream approximately 1 m deep, using electrofishing gear and hand nets against the current over a sandy substrate (Figure 7). Additional paratypes were also captured during the day in similar microhabitats (i.e., sandy bottom) using a seine net and by actively disturbing the substrate. Sampling sites ranged from 15 to 20 m above sea level.

The following species were caught with the new species: *Acentronichthys leptos* Eigenmann & Eigenmann, 1889, *Ancistrus multispinis* (Regan, 1912), *Atlantirivulus haraldsiolii* (Berkenkamp, 1984), *Awaous tajasica* (Lichtenstein, 1822), *Characidium pterostictum* Gomes, 1947, *C. lanei* Travassos, 1967, *Deuterodon iguape* Eigenmann, 1907, *Hyphessobrycon* sp., *Hisonotus leucofrenatus* (Miranda Ribeiro, 1908), *Hollandichthys multifasciatus* (Eigenmann & Norris, 1900), *Homodiaetus graciosa* Koch, 2002, *Kronichthys lacerta* (Nichols, 1919), *Mimagoniates microlepis* (Steindachner, 1877), *Scleromystax barbatus* (Quoy & Gaimard, 1824), *Schizolecis guentheri* (Miranda Ribeiro, 1918), *Synbranchus marmoratus* Bloch, 1795, *Pimelodella transitoria* Miranda, Ribeiro 1907, *Phalloceros pellos* Lucinda, 2008, *P. alessandrae* Lucinda, 2008, *P. titthos* Lucinda 2008, *Rhamdia quelen* (Quoy & Gaimard, 1824), *Rhamdioglanis frenatus* Ihering, 1907 and *Rineloricaria kronei* (Miranda Ribeiro, 1911).

#### 3.1.9 Conservation status

The Guaraqueçaba basin landscape has undergone significant historical modifications. A decade ago, nearly 5% of the Guaraqueçaba Environmental Protected Area (EPA) landscape had been altered due to the expansion of pasture grassland areas and extraction of sand (Kauano et al., 2012; Souza et al., 2020). *Microcambeva caissara* is apparently a rare species only known from two nearby localities in the Guaraqueçaba EPA, which represent a single location as defined in the IUCN (2019) guidelines. Therefore, we recommend that *M. caissara* be classified as vulnerable (VU) in accordance to the criterion D2 of the International Union for Conservation of Nature (IUCN, 2019).

### 3.2 *Microcambeva ribeirae* Costa, Lima & Bizerril, 2004

urn:lsid:zoobank.org:act:EA9A5362‐891F‐416D‐964B‐89A189A9FC47.

**FIGURE 8:**
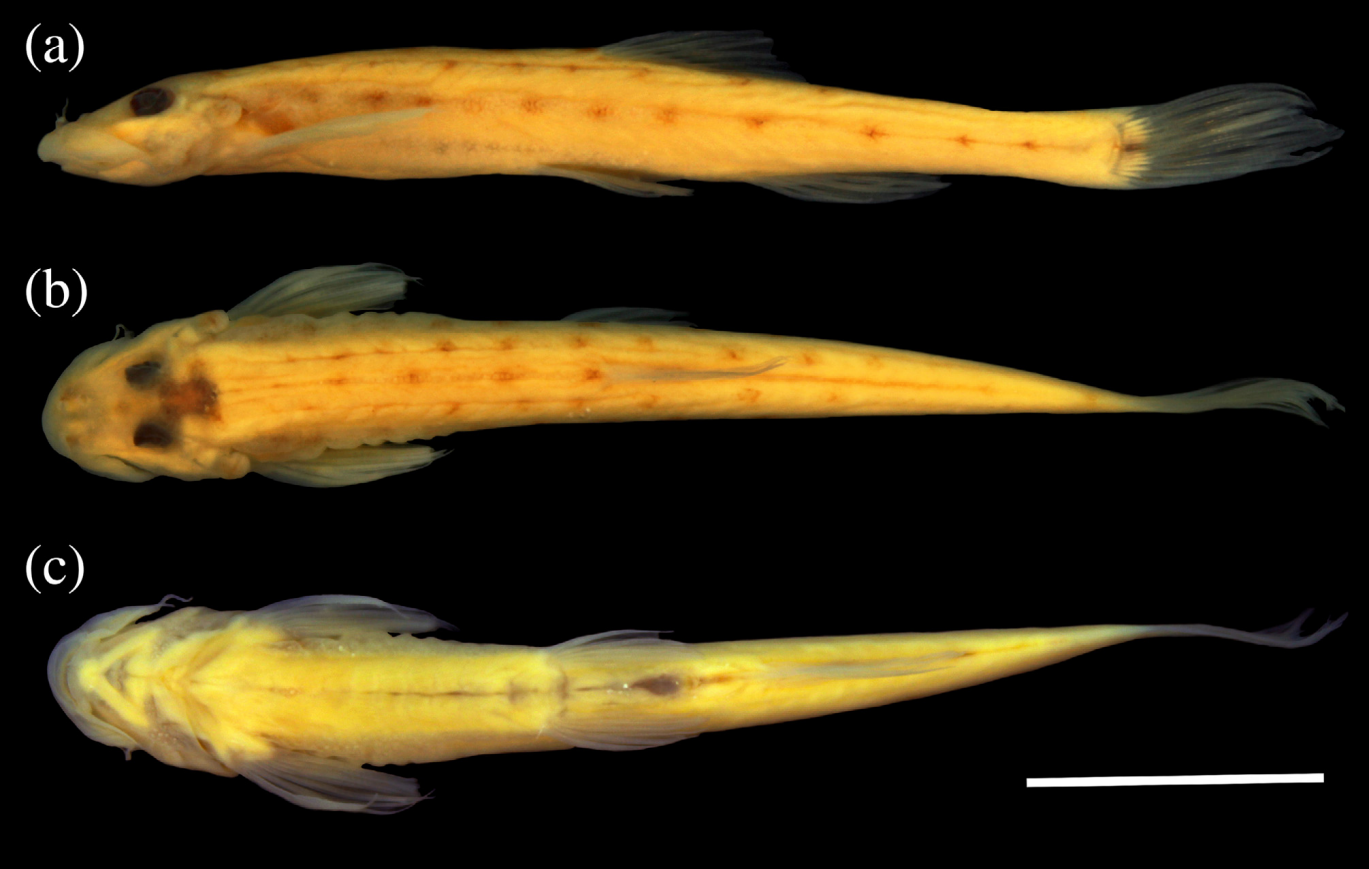
*Microcambeva ribeirae*, holotype, MZUSP 84301, 47.8 mm *L*_S_, São Lourencinho River, Ribeira de Iguape River basin, Pedro Toledo, São Paulo, Brazil: (a) left lateral view, (b) dorsal view, (c) ventral view. Scale bar: 10 mm.

**FIGURE 9:**
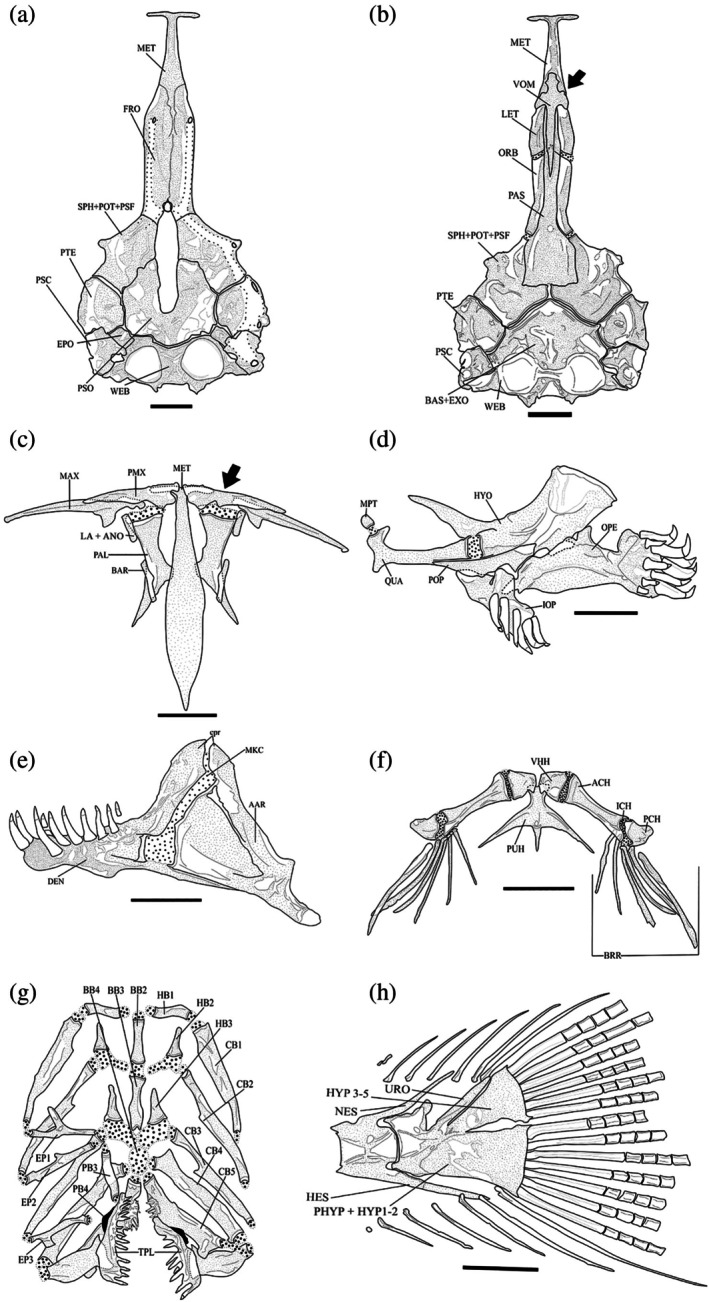
Skeleton of *Microcambeva ribeirae*, MZUPS 65764, 40.7 mm *L*_S_ non‐type series. Skull and Weberian apparatus in (a) dorsal and (b) ventral view. (c) Dorsal view of upper jaw and associated structures. (d) Suspensorium and opercular apparatus, left side, lateral view, (e) lower jaw, left side, external view, (f) hyoid arch, ventral view, (g) branchial skeleton, ventral view, (h) caudal skeleton, left side, ventral view. AAR, anguloarticular‐retroarticular; ACH, anterior ceratohyal; BAR, barbular; BAS + EXO, basioccipital + exoccipital; BB2‐4, basibranchials 2–4; BRR, branchiostegal rays; CB1‐5, ceratobranchials 1–5; cpr, coronoid process; DEN, dentary; EPO, epioccipital; EP1‐4, epibranchials 1–4; FRO, frontal; HB1‐3, hypobranchials 1–3; HES, haemal spine; HYO, hyomandibula; HYP 1–2 + PH, hypurals 1–2 + parahypural fused; HYP 3–5, hypurals 3–5 fused; ICH, interceratohyal cartilage; IOP, interopercle; LA + ANT: Lacrimal + antorbital LET, lateral ethmoid; MAX, maxilla; MET, mesethmoid; MKC, meckel's cartilage; MPT, metapterygoid; NES, neural spine; OPE, opercle; ORB, orbitsphenoid; PAL, palatine; PAS, parasphenoid; PB3‐4, pharyngobranchials 3–4; PCH, posterior ceratohyal; PHYP, parahypural; PMX, premaxilla; POP, preopercle; PSC, posttemporo‐supracleithrum; PSO, parieto‐supraoccipital; PTE, pterotic; PUH, parurohyal; QUA, quadrate; SPH + POT + PSF, sphenotic + prootic + pterosphenoid complex; TPL, toothplate; URO, uroneural spine; VHH, ventral hypohyal; VOM, vomer; WEB, capsule of weberian apparatus. Black arrows indicate small lateral pointed expansions in median lateral portions of anterior portion of vomer (b) and lateral process of premaxilla long, approximately twice of length of the bone, and pointed (c). Scale bar: 1 mm, except for lower jaw, which is 0.5 mm.

**FIGURE 10:**
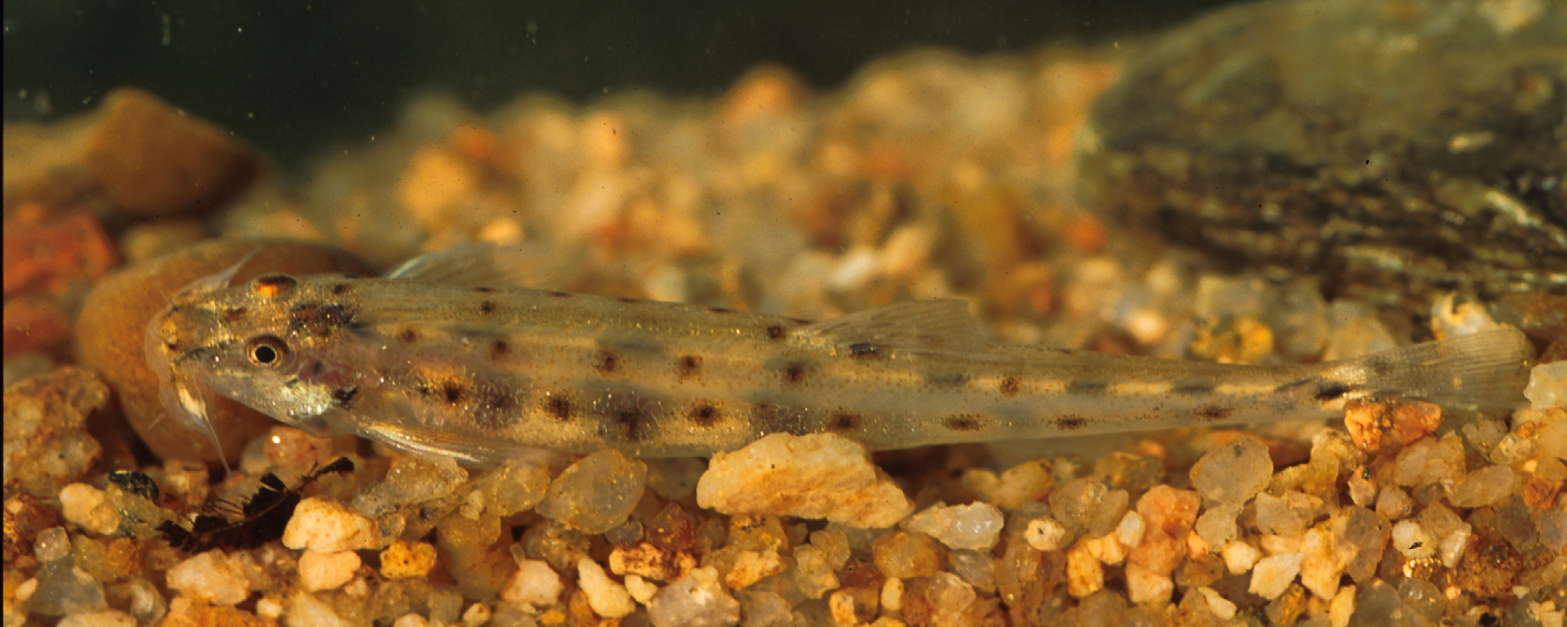
Live specimens of *Microcambeva ribeirae*, MZUSP 116750, 45.7 mm *L*_S_, Brazil, São Paulo State (photo by Osvaldo Oyakawa).

Figures 8, 9, 10 and Table 3.

*Microcambeva ribeirae* Costa et al., 2004 (original description). Wosiacki & Oyakawa, 2005: 470 (list); Oyakawa et al., 2006: 113 (description of habitat, geographic distribution, taxonomic notes); Zuanon et al., 2006: 115 (ecological notes, psammophilic habits); Ferraris, 2007: 409 (list); Menezes et al., 2007: 303 (description of habitat, geographic distribution); Lima et al., 2008: 321 (comparative material); Claeson et al., 2008: 4, (description of latero‐sensory pores); Mattos & Lima, 2010: 238 (comparative material); Datovo & Bockmann, 2010: 197, 208, 219 (comparative material, myological description, character matrix); Wosiacki et al., 2011: 1 (colour pattern); Oyakawa & Menezes, 2011: 28 (list); Gonçalves & Braga, 2013: 183 (list); Lima et al., 2013: 522 (comparative material); Villa‐Verde et al., 2013: 645 (comparative material); Datovo et al., 2016: 453 (comparative material); Castro & Wosiacki, 2017: 2014 (morphological comparations and comparative material); Andrade et al., 2017: 443 (list); Montoya‐Ospina et al., 2018: 101 (list); Pupo & Britto, 2018: 9 (comparative material); Costa et al., 2019: 1841, 1844 (comparative material); Sarmento‐Soares et al., 2019: 585 (taxonomic notes, geographic distribution and conservation); Costa et al., 2019: 62 (ecological notes); Costa et al., 2019: 5, 7 (phylogenetic relationship and osteological notes); Medeiros et al., 2020: 121 (comparative material); Costa, Henschel, & Katz, 2020: 13, 14 (comparative material); Costa, Henschel, & Katz, 2020: 82 (ecological notes); Costa, Vilardo, & Katz, 2020: 127 (phylogenetic tree); Ferrer & Malabarba, 2020: 72 (comparative material); Cetra et al., 2020: 10 (geographic distribution); Vilardo et al., 2021: 1094 (phylogenetic relationships); Medeiros et al., 2021: 12 (comparative material); Costa & Katz, 2021: 318, 321, 322, 323, 331, 332, 341 (phylogenetic relationships, osteological description of mesethmoid region and adjacent bones, left jaw suspensorium and opercular series, and character matrix); Costa et al., 2021: 5, (phylogenetic tree); Costa, 2021: 183 (comparative material, character matrix); Cetra et al., 2022; 10 (geographic distribution); Costa et al., 2022, 495: (phylogenetic relationships); Costa et al., 2023, 5: (phylogenetic relationships).

#### 3.2.1 Diagnosis

*Microcambeva ribeirae* is distinguished from all congeners by having small lateral pointed expansions in median lateral portions of anterior portion of vomer (vs. expansions absent). It differs from *M. filamentosa* by having first pectoral‐fin ray not extended into filament (vs. prolonged as filament). It differs from *M. bendego* by tips of rictal barbels reaching anterior region of interopercular odontodophore (vs. reaching middle of orbit), and presence of two unbranched dorsal‐fin rays (vs. three). It differs from *M. caissara* by having 34 vertebrae (vs. 32), maxillary barbel tip reaching beyond posterior portion of interopercular odontodophore (vs. reaching anterior portion of interopercular odontodophore).

#### 3.2.2 Redescription of *Microcambeva ribeirae*

Morphometric data of holotype, type series and additional specimens in Table 3. Body elongated, head wider than trunk in dorsal view (Figure 8a,c). Cross‐section of body cylindrical posterior to head, progressively compressed at pelvic‐fin insertion, tapering to caudal fin. In lateral view, lowest body depth posterior to head, deepest approximately at dorsal‐fin origin. Dorsal body profile slightly convex from tip of snout to dorsal‐fin origin; straight along caudal peduncle, slightly concave at dorsal procurrent rays. Ventral profile of body convex, from gular region to insertion of pectoral fin; straight between anus and caudal peduncle; slightly concave at ventral procurrent rays.

Head depressed, triangular in dorsal view, longer than wide, less deep than body (Figure 8b). Mouth subterminal, wide; upper jaw longer than lower (Figure 8c). Barbels large; similar to each other in general aspect, their internal cores visible by transparency. Anterior nostril small, round, located closer to upper lip than to anterior margin of eye, surrounded by small tube of integument continuous posterolaterally with nasal barbel. Posterior nostril round, larger than anterior one, situated slightly closer to eye than to anterior nostril, surrounded by low rim of integument. Maxillary barbel, originating laterally at upper lip; wide at base, progressively tapering to fine distal tip reaching median portion between interopercular odontodophore (*n* = 27, including holotype), anterior portion of interopercular odontodophore (*n* = 10) or posterior portion of opercular odontodophore (*n* = 6). Rictal barbel originating laterally at lower lip, tips reaching anterior portion of interopercular odontodophore (*n* = 42, including holotype) or, in rare cases, median portion of interopercular odontodophore (*n* = 1). Pair of finger‐like barbels (sensu Medeiros et al., 2020) approximately half diameter of eye, set closely to branchial isthmus. Nasal barbel originating at latero‐median portion of anterior nostril, wide at base, tapering distally, reaching posterior margin of posterior nostril (*n* = 28, including holotype), anterior region of posterior nostril (*n* = 12) or, in rare cases, anterior margin of eye orbit (*n* = 3). Eye round, wide, covered by thin, transparent skin, located dorsolaterally on head. Interorbital of same size as eye diameter. Branchiostegal membranes narrowly united to isthmus. Five pleural ribs. Vertebrae 34 (28 caudal, six pre‐caudal).

Pectoral‐fin rays i + 6 (*n* = 43, including holotype). First ray unbranched, not prolonged as filament, approximately half as long as first branched ray, remaining pectoral‐fin rays branched, progressively longer than membrane, tips of rays forming continuous line along fin margin (Figure 8b). Axillary gland, located dorsoposteriorly to pectoral‐fin insertion. Dorsal fin ii + 6 + i (*n* = 43, including holotype), its origin at vertical through 14th vertebrae. Pelvic fins i + 4 (*n* = 42, including holotype) or i + 6 (*n* = 1), its origin at vertical through 10th vertebrae. Bases of pelvic fins separated from each other, their margin covering urogenital papilla. Urogenital papilla conical. Anal fin iii + 4 (*n* = 43, including holotype), larger than dorsal fin, originating at vertical through 19th vertebrae. Caudal fin truncate, i + 11 + i or i + 10 + ii; six principal rays (i + 5) on dorsal and seven (6 + i) on ventral hypural plate. Dorsal and ventral procurrent caudal‐fin rays six, with ventral larger than dorsal ones.

Mesethmoid long; main body of bone compressed at posterior portion, main axis and anterior margin straight; posterior portion tapering, overlapped posterodorsally by frontal (Figure 8a). Mesethmoid cornua short, straight, with pointed tips. Frontal slender, short. Cranial fontanel round anteriorly and posteriorly. Sphenotic, pterosphenoid and prootic fused continuous bone. Vomer long, arrow‐shaped; its anterior portion small, with rounded dorsal margin, lateral constriction at anterior portion, minute lateral projections anterior to constriction with tips directed posteriorly; broadest portion of vomer at lateral projections; posterior portion long, pointed (Figure 8b). Lateral ethmoid rectangular, without lateral projections. Parasphenoid narrow anteriorly, wide posteriorly; anterior extremity bifurcated around vomer; posterior extremity straight. Basioccipital fused with exoccipital and Weberian complex.

Premaxilla triangular, with narrow, elongated, toothless lateral process about twice of premaxilla length. Conspicuous protuberance on dorsal surface at posteromedial portion of premaxilla near palatine cartilage (Figure 9c). Premaxillary teeth 12–15 in labial row, 11–13 in lingual. Dentary triangular, with two rows of small conical teeth, 10–11 in external row, 6–10 in internal row (Figure 8e). Maxilla, narrow, elongate with pointed tips; 50% longer than premaxilla (including lateral process). Palatine long, with posterolateral elongated, pointed process nearly as long as remainder of palatine; process straight, its lateral margin with pronounced concavity. Lacrimal‐antorbital cylindrical, elongated, approximately 65% as long as barbular length. Barbular cylindric, elongate.

Metapterygoid elliptic (Figure 9d). Quadrate elongate, with small pointed process extending anterodorsally from anterior tip, its dorsal margin slightly concave, gradually curving posteriorly. Hyomandibula with narrow, pointed, elongated anterior process, curved ventrally, reaching anterior tip of preopercle. Preopercle straight, as long as quadrate, tapering anteriorly; posterior end roundish. Interopercle with 6–9 conical odontodes, disposed obliquely, arranged in two irregular rows. Opercle slender, with pronounced dorsomedial concavity, small pointed process anteriorly. Opercular odontodes 9–14, conical, arranged obliquely in three or four irregular rows. Opercle with large, pointed ascending process. Dentary with two rows of small conical teeth, 10–11 in external row, 6–10 in internal row (Figure 8e).

Parurohyal with central, process shorter than lateral wings (Figure 9f). Central parurohyal foramen small, ellipsoid. Ventral hypohyal trapezoid, with deep fossa on ventral surface for articulation with parurohyal condyle. Anterior ceratohyal long, rod‐like, constricted at middle. Posterior ceratohyal sub‐triangular. Branchiostegal rays 6. Basibranchial 1 absent. Basibranchial 2 and 3 rod‐shaped. Basibranchial 4, fully cartilaginous, large, roundish, depressed (Figure 9g). Hypobranchial 1 rod‐shaped. Hypobranchial 2 and 3 conical. Ceratobranchials 1 to 4 cylindrical. Ceratobranchial 1 and 2 with small triangular expansion at ventromedial region; ceratobranchial 4 with triangular expansion at antero‐dorsal margin; ceratobranchial 5 rectangular, excluding ventroposterior process, with five or six conical pharyngeal teeth irregularly arranged at mesial margin. Epibranchial 1 Y‐shaped. Epibranchial 2 with two small lateral expansions: one at middle of dorsal surface, directed anteriorly, another antero‐ventral directed posteriorly. Epibranchial 3, rod‐shaped, with laminar expansion distally at antero‐ventral portion (resembling an axe). Pharyngobranchials 1 and 2 absent; pharyngobranchial 3 rod‐chaped; pharyngobranchial 4 cartilaginous, inserted laterally at antero‐dorsal margin of upper lower pharyngeal tooth plate. The latter, bearing 12 or 14 conical teeth in single row.

Hypural complex with two plates. Ventral plate rectangular, formed by fusion of parhypural plus hypurals 1 and 2; dorsal plate formed by fusion of hypurals 3 to 5 (Figure 9h). Uroneural elongate, slightly shorter than adjacent neural spine, not fused with dorsal hypural plate.

#### 3.2.3 Colour in ethanol

Dorsal, lateral and ventral surfaces of body yellowish pale (Figure 8). Dorsal series with four or five spots irregularly spaced, first spot posterior to occipital region, fourth or fifth spot at dorsal‐fin insertion, sixth spot at final portion of caudal peduncle. Upper‐lateral series with seven or eight spots. Mid‐lateral series with 9–13 unevenly‐spaced spots; first one dorsal to pectoral‐fin base; last one largest, ellipsoid, covering hypural plate and central region of caudal‐fin base. Posterior portion of dorsal surface of head with large dark mark roughly on posterior surface of neurocranium, extending from area slightly anteriorly to line through posterior margin of eyes to anterior limit of epaxial musculature, ‘hourglass‐shaped’. Mark formed by combination of brain pigment and paired integumentary dark spots positioned posteriorly. Slender dark field extending between anterior and posterior nostrils. Remainder of dorsal surface of head with uniform covering of light brownish chromatophores. Intensely dark concentration of chromatophores on opercular odontodophore. Ventral surface of head white. Maxillary and nasal barbels with small dark chromatophores dispersed at base. Base of barbels yellowish, white along the thinner portion. Greyish vertical bar across caudal‐fin base.

#### 3.2.4 Colour in life

Dorsal and trunk almost translucent, ventral surface of body yellowish, pale. Series of large dark spots distributed along body (Figure 10). Large spots brown circling small black spots. Small melanophores distributed irregularly on body. Mid‐lateral, upper lateral and mid‐dorsal series of spots similar to those observed in preserved specimens. Head with large dark blotch extending over middle of posterior part of neurocranium. Eyes black, iris orange. Ventral surface of head uniformly yellowish. Slender brownish blotch extending laterally between anterior and posterior nostrils. Base of maxillary barbel silvery; distal portion of barbels hyaline. First pectoral‐fin ray silvery. Fins hyaline. Grey vertical bar across caudal‐fin base.

#### 3.2.5 Geographic distribution and ecology

*Microcambeva ribeirae* occurs in the upper and middle portions in the Ribeira de Iguape basin, in the Ribeira de Iguape ecoregion (Figure 6). Most sampling sites are in small tributaries of the main channel, characterized by clear water, weak current, substrate composed mostly of sand, clay and a few stones, average depth of 0.5–1 m and width of 2–4 m. Most localities are about 40 m above sea level, with extremes varying between 5 and 280 m. The climate of the region is classified as Cfb in the Köppen scale, featuring subtropical highland climate (Peel et al., 2007).

#### 3.2.6 Conservation status

The species is found in the most extensive area of Atlantic Forest remaining in São Paulo State (Cetra et al., 2020). It was recorded in four protected areas: the Serra do Mar, Cananéia‐Iguapé‐Péruibe and Quilombos do Médio Ribeira EPAs, and the Serra do Mar State Park (Cetra et al., 2020; Sarmento‐Soares et al., 2019). The species is frequently collected, but not abundant in the sampling sites. Its extent of occurrence (EOO) is less than 20,000 km^2^ (~2,600 km^2^), and the extinction threats to the species are diffuse, mostly related to agricultural activities and extraction of sand. While these factors directly impact some populations of *M. ribeirae*, they do not pose an imminent risk of extinction. In this context, we agree with the inclusion of the species as the ‘least concern (LC) category, as proposed by Netto‐Ferreira et al. (2024).

#### 3.2.7 Phylogenetic analysis

**FIGURE 11:**
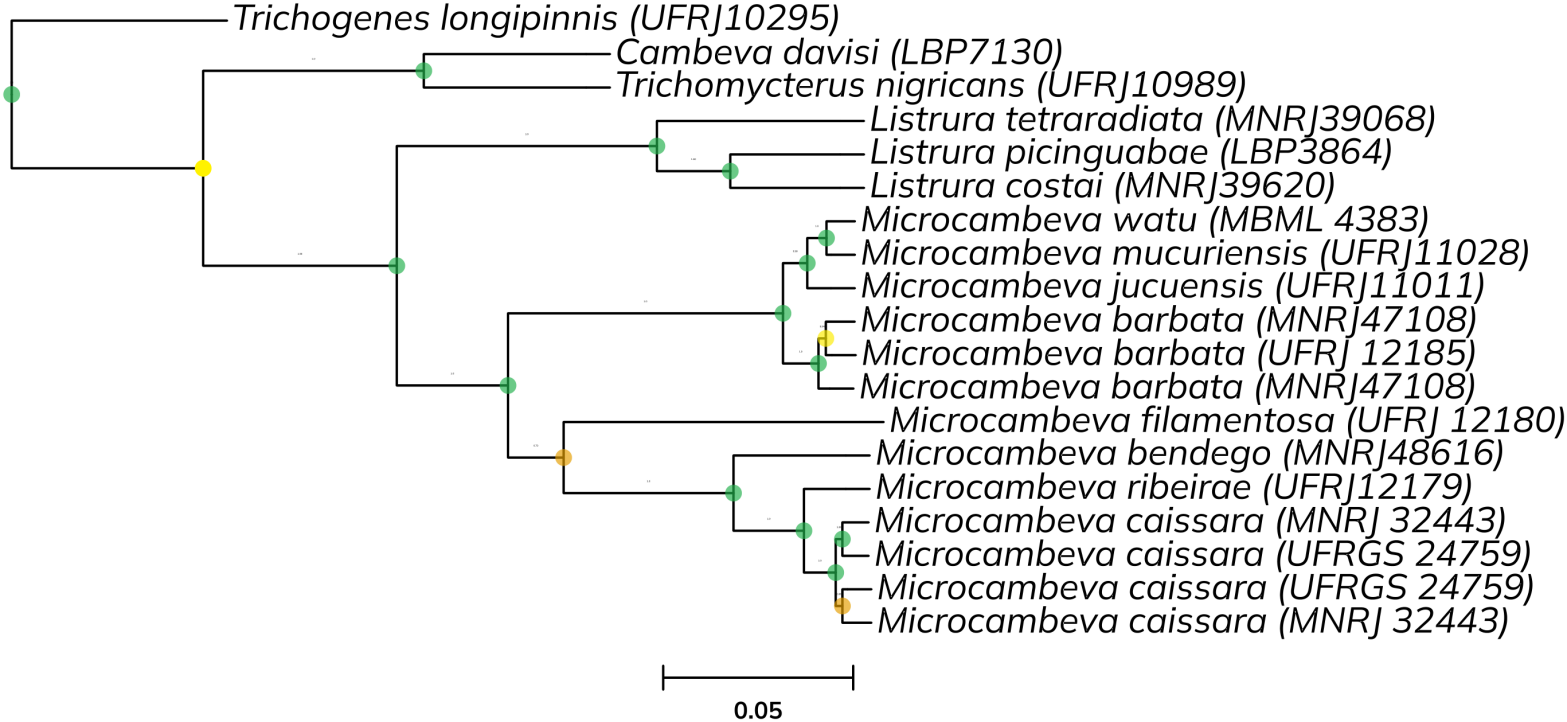
Bayesian inference phylogenetic tree of *Microcambeva* species (*M. draco* not included) and outgroup taxa based on mitochondrial gene cytochrome c oxidase subunit I (*cox1*). Coloured circles in the nodes correspond to posterior probability (PP) values. Orange circle, ≤0.75 PP; yellow circle, ≤0.95 PP; green circle, 1.0 PP.

**FIGURE 12:**
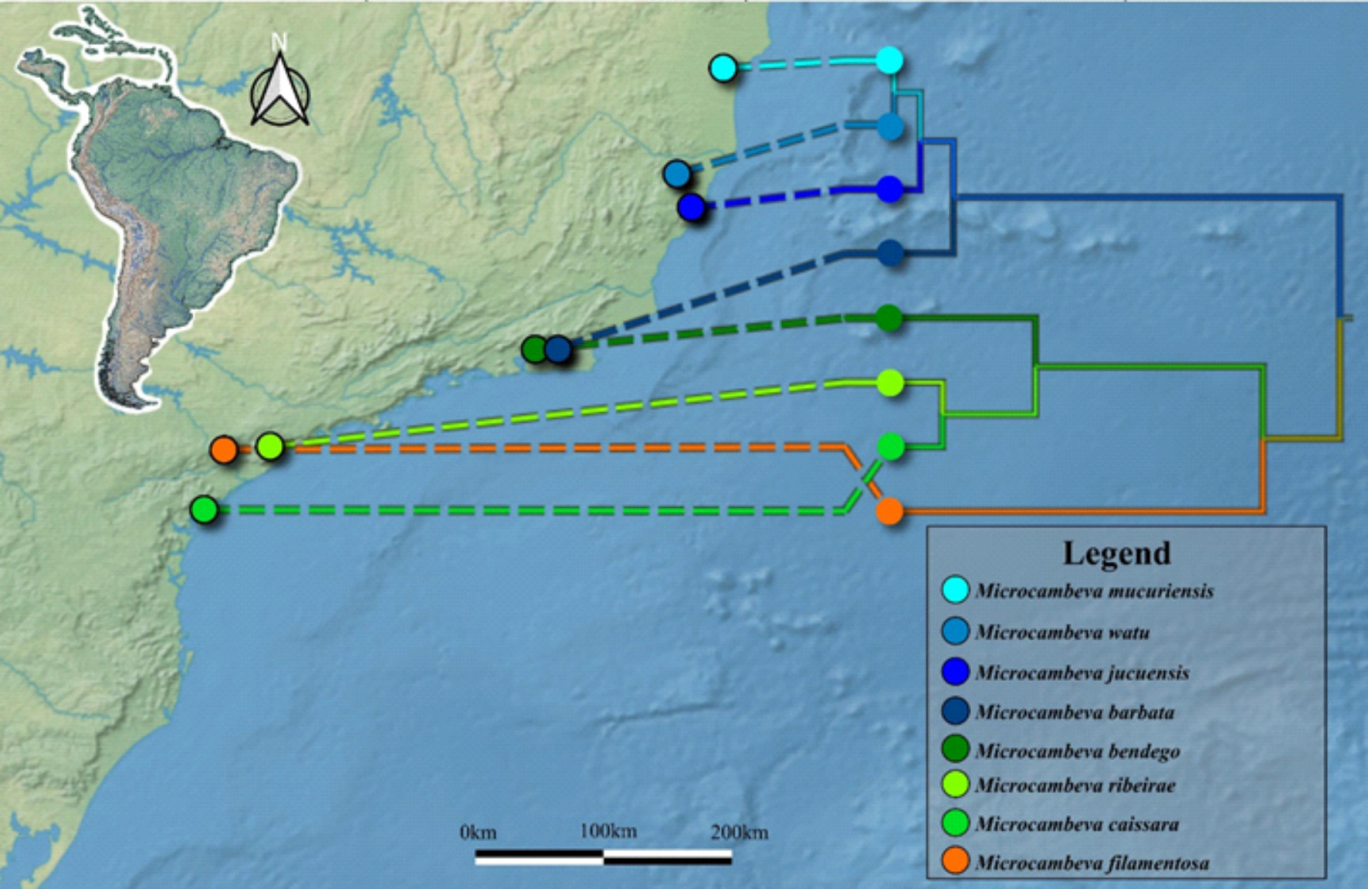
Geophylogeny of the *Microcambeva* genus (species *M. draco* not included) derived from the BI phylogenetic sub‐set tree for cytochrome c oxidase subunit 1 (*cox1*). Taxa were mapped to type localities.

According to the BI tree of the *cox1* gene, *M. caissara* is the sister species of *M. ribeirae* (posterior probability = 1.0) and those two species form a monophyletic group with *M. bendego* (Figures 11 and 12). The BI analysis also revealed a close relationship with other species of *Microcambeva* occurring southward of the northeastern river basins divides of the Macacu basin. The average pairwise genetic distance between *M. caissara* and *M. ribeirae* is 2.2% (Table 4). Interspecific distances are greater to *M. bendego* (6.7%, 6.9%), *M. filamentosa* (16.0%, 17.7%) and other species in the genus (17% to 18%).

**TABLE 4:** Values of pair‐wise mtDNA genetic distance values for cytochrome c oxidase subunit 1 (*cox1*) gene between and within *Microcambeva* species using a Kimura 2 parameter model.

	1	2	3	4	5	6	7	8
1. *M. caissara* (*n* = 4)	–							
2. *M. ribeirae* (*n* = 1)	0.022	–						
3. *M. bendego* (*n* = 1)	0.065	0.065	–					
4. *M. filamentosa* (*n* = 1)	0.147	0.142	0.155	–				
5. *M. barbata* (*n* = 3)	0.160	0.158	0.158	0.156	–			
6. *M. watu* (*n* = 1)	0.163	0.155	0.170	0.163	0.023	–		
7. *M. mucuriensis* (*n* = 1)	0.163	0.155	0.170	0.163	0.025	0.002	–	
8. *M. jucuensis* (*n* = 1)	0.163	0.156	0.171	0.160	0.014	0.010	0.012	–

## 4 DISCUSSION

The use of molecular and morphological approaches in taxonomic studies of Neotropical fishes provides integration of independent lines of evidence in species recognition and phylogenetic studies (Albornoz et al., 2024; Malabarba et al., 2021; Medeiros et al., 2024; Reis et al., 2020; Reis & de Pinna, 2023; Villa‐Verde et al., 2013). By integrating these approaches, we were able to gather compelling evidence supporting the description of *M. caissara* based on specimens from the Guaraqueçaba River basin, which were identified as *M. ribeirae* in previous studies (Costa & Katz, 2021; Medeiros et al., 2021; Sarmento‐Soares et al., 2019). It also allowed us to establish the taxonomic boundaries between *M. ribeirae* and *M. caissara*, and to establish the position of *M. bendego* in the *Pterocambeva* subgenus and *M. watu* in the Microcambeva subgenus based on DNA sequence data, corroborating a hypothesis previously established based solely on morphological data (Costa & Katz, 2021).

Morphological differences among species of *Microcambeva* are primarily associated with osteological features of the mesethmoid and opercular regions, along with subtle differences in qualitative and meristic characters (Costa et al., 2019; Costa, Vilardo, & Katz, 2020; Medeiros et al., 2020, 2021). In the opercular apparatus, the anterior process of the opercle is longer in *M. caissara* (Figure 3d) than in *M. bendego* (Medeiros et al., 2020: Figure 3). In the mesethmoid region, the lateral process of the maxilla is short and blunt in *M. caissara* but elongated and pointed in *M. ribeirae* (Figure 8). Additionally, coloration of the cephalic dorsal pigmentation in fixed specimens is an easily observable external feature that can be used to diagnose the species, as proposed by Medeiros et al. (2021). The dorsal pigmentation on the cephalic region in *M. caissara* forms a T‐shaped mark, whereas in *M. ribeirae* it is hourglass‐shaped and in *M. bendego* it forms a poorly‐defined hourglass (Figure 1b).

Here, the phylogenetic placement of *M. watu* and *M. bendego* was determined based on the *cox1* gene, whereas Costa and Katz (2021) inferred their relationship based solely on morphological data. The pairwise genetic distances (K2P) among *Microcambeva* species reveal that species of the same subgenus exhibit low genetic distances, while higher values are observed between species from different subgenera. The pairwise genetic distances (K2P) among species of Microcambevinae confirms that a 2% divergence in the mtDNA *cox1* is the minimum interspecific value for distinguishing nominal species, as observed by Villa‐Verde et al. (2013) and Medeiros et al. (2024), and is effective in delimiting species within the *Pterocambeva* subgenus. However, it is imperative to examine these molecular data meticulously, as even pairwise genetic distances inferior to 2% can simultaneously indicate distinct species (Table 4), as observed a genetic distance of 0.2% between *M. watu* and *M. mucuriensis*, and 0.1% of the former one species and *M. jucuensis*. At the same time, the genetic distance between *M. jucuensis* and *M. barbata* is 0.1%, while between *M. jucuensis* and *M. mucuriensis* is 0.2%.

The Ribeira de Iguape and Guaraqueçaba are coastal basins with headwaters separated by a few kilometres on the slopes of the Serra do Mar mountains, and intervening headwaters of the Cananéia River system and the Serra Negra River (Figure 6). According to paleodrainage reconstructions by Thomaz and Knowles (2018), based on a scenario of ~150 m sea level retreat during the Pleistocene glaciation, the paleodrainages of the aforementioned rivers were not connected during that geologic period. If so, hypothetical headwater capture events could explain the occurrence of sister lineages in different slopes of a mountain range. Ribeiro (2006) proposes that the eastern margin of the Brazilian Shield has undergone significant geologic activity during the Miocene and Pliocene, which reactivated ancient faults, and resulted in multiple headwater capture events between adjacent drainages. However, any biogeographic hypothesis would require further investigation of the ichthyofauna of the Serra Negra and Cananéia watersheds to determine the presence of populations of *Microcambeva* in these basins.

Previously, *M. ribeirae* was categorized as ‘near threatened’ in the list of threatened fauna of São Paulo State (Oyakawa et al., 2009), while in the recent Brazilian Red List the species was categorized as ‘least concern’ (LC) (Netto‐Ferreira et al., 2024). Here we exclude the Guaraqueçaba basin from the geographic distribution of *M. ribeirae*, which is limited to the middle and upper portions of the Ribeira de Iguape basin, where it has been recorded in four conservation units and is relatively common but not abundant (Netto‐Ferreira et al., 2024; Sarmento‐Soares et al., 2019). The anthropogenic alterations in the Ribeira de Iguape River basin are limited, primarily associated with agricultural activities and localized pollution. These threats are diffuse and do not pose an imminent risk of extinction on the species (Netto‐Ferreira et al., 2024). In contrast, *M. caissara* is known from only two localities, distant approximately 13 km, one of which is situated in the Salto Morato EPA, suggesting a restricted distribution. Considering the diffuse anthropogenic impacts (Kauano et al., 2012), the dynamics of human occupation in the region and the environmental sensitivity of lowland forests, we suggest that *M. caissara* be classified as ‘vulnerable’ (VU) according to criterion D2 of the International Union for the Conservation of Nature (IUCN, 2019).

In species with restricted distribution ranges, limited dispersal capabilities, small population sizes and minimal gene flow, the fixation of novel alleles may play a significant role in the process of allopatric speciation (Avise, 2000). Integrating morphological and genetic evidence that encompass almost all species of *Microcambeva*, we provided evidence of the taxonomic validity of *M. caissara* and *M. ribeirae*. An update on the geographic distribution and conservation status is provided for *M. ribeirae*, a species restricted to the Ribeira de Iguape basin. The description of *M. caissara*, only known from the Guaraqueçaba basin, contributes to the knowledge of the fish fauna of the coastal basins of the Atlantic Forest, and demonstrates that there is an overlooked hidden diversity of trichomycterids in poorly collected sandy environments.

## 5 EXAMINED MATERIAL

### 5.1 Microcambeva ribeirae

MZUSP 84301, 47.7 mm *L*_S_, Holotype, São Lourencinho River, Pedro Toledo; MZUSP 78617, 5, 40.2–47.6 mm *L*_S_, same locality of holotype, MZUSP 79953, 7, 25.8–35.6 mm *L*_S_, Espraiado River, Iguape, MNRJ 14304, 3, 29.4–32.3 mm *L*_S_, Areado stream, Miracatu, MZUSP 116104, 4, 33.1–36.5 mm *L*_S_, Espraiado River, Iguape, MZUSP 84381, 3, 38.1–41.7 mm *L*_S_, Ipiranga River, Sete Barras, MZUSP 74699, 10, 32.3–47.4 mm *L*_S_, Fau River, Miracatu, MZUSP 69405, 2, 45.8–45.9 mm *L*_S_, Fau River, Miracatu, MZUSP 50588 2, 32.8–35.8 mm *L*_S_, Serra streams, Miracatu, MNRJ 37165, 1, 40.5 mm *L*_S_, Jacupiranga Riber, Jacupiranga, MZUSP 69425, 5, 38.3–40.6 mm *L*_S_, Ipiranga River, Sete Barras, MZUSP 65765, 3 39.7–41.1 mm *L*_S_, Ipiranga River, Sete Barras, MZUSP 65766, 1, 36.4 mm *L*_S_, Quilombo River, Sete Barras, MZUSP 65764, 4 (4C&S), 30.7–40.7 mm *L*_S_, Preto River, Sete Barras.

### 5.2 Microcambeva bendego

Guapi‐Macacu River system: MNRJ 52042, 28.1 mm *L*_S_, Holotype, Guapiaçu River near Cachoeiras de Macacu, Guapimirim, MNRJ 48616, 1, 26.9 mm *L*_S_, MZUSP 125789, 1, 27.8 mm *L*_S_, same locality of holotype.

### 5.3 Microcambeva watu

Doce River basin: MNRJ 51962, 25.7 mm *L*_S_, Holotype, stream between São João de Petrópolis and Vila 25 de Julho, Santa Teresa, MBML 4400, 3 (1C&S) 19.5–22.1 mm *L*_S_, MBML 4383, 1, 26.0 mm *L*_S_, UFRN 5800, 2 (1 C&S), 21.8–20.8 mm *L*_S_, same locality of holotype, MZUSP 123346, 1, 23.9 mm *L*_S_, Corrente Grande River, Penha, MZUSP 123363, 2, 26.8–27.3 mm *L*_S_, Corrente Grande River, Penha, MZUSP 123364, 4, 20.6–22.7 mm *L*_S_, MZUSP 123365, 6 (3 C&S), 23.0–23.9 mm *L*_S_, Ribeirão São Mateus, Baguari.

### 5.4 Microcambeva jucuensis

UFRJ 12124, 27.2 mm *L*_S_, Holotype, stream close to Nova Campo Grande village, Viana, UFRJ 11083, 18, 19.3–27.1 mm *L*_S_, UFRJ 11011, 4, 19.1–22.3 mm *L*_S_, UFRJ 11840, 4 (C&S), 21.1–22.0 mm *L*_S_, same locality of holotype. MZUSP 91641, 11, 19.5–24.6 mm *L*_S_, Formate River, in Nova Campo Grande, Viana, UFRGS 13058, 1, 25.42 mm *L*_S_, Formate River, near Viana city, Viana.

### 5.5 Microcambeva mucuriensis

UFRJ 12123, 25.2 mm *L*_S_, Holotype, Mucuri River, Bahia, UFRJ 11091, 22, 14.5–19.3 mm *L*_S_, UFRJ 11028, 4, 14.2–17.2 mm *L*_S_, UFRJ 11841, 5, 15.3–19.05 mm *L*_S_, same locality of holotype, MZUSP 55195, 16.66 mm *L*_S_, Tributary of Mucuri River, Nanuque.

### 5.6 Microcambeva draco

MCP 17796, 24.6 mm *L*_S_, Holotype, Jucuruçu River, Itamaraju, MCP 47695, 1, 24.1 mm *L*_S_, MCP 47695,1, 24.1 mm *L*_S_, same locality of holotype, MCP 36634, 11, 17.2–20.3 mm *L*_S_, Tributary of Fazenda River, Caravelas.

### 5.7 Microcambeva barbata

MZUSP 43678, 26.1 mm *L*_S_, Holotype, São João River, near Gaviões, Silva Jardim, MZUSP 43679, 21.4 mm *L*_S_, same locality of holotype, DZSJRP 13861, 1, 25.14 mm *L*_S_, Tributary of the São João River, Silva Jardim, MNRJ 37572, 1, 26.33 mm *L*_S_, Aldeia Velha stream, Casimiro de Abreu, MNRJ 4708 19.59 mm *L*_S_, 1, Aldeia Velha River, Saquarema, MNRJ 49371, 1, 22.69 mm *L*_S_, Aldeia Velha River, Casimiro de Abreu, MNRJ 50998, 2, 25.99–22.08 mm *L*_S_, Aduelas River, Macaé, MZUSP 79828, 2, 26.4–24.6 mm *L*_S_, São João River, Silva Jardim, MZUSP 80225, 10, 19.6–25 mm *L*_S_, Tributary of São João River, Silva Jardim.

### 5.8 Microcambeva filamentosa

UFRJ 12187, 31.4 mm *L*_S_, Holotype, Preto River, Sete Barras, UFRJ 12180, 2; 25.6–28.1 mm *L*_S_, UFRJ 12188, 1, 30.4 mm *L*_S_, UFRJ 12345, 2, 30.8–31.6 mm *Ls*, UFRJ 12551, 9, 30.7–35.3 mm *L*_S_, same locality of holotype.

## AUTHOR CONTRIBUTIONS

**Lucas S. de Medeiros**: Conceptualization; methodology; software; validation; formal analysis; investigation; data curation; writing—original draft; visualization, and supervision. **Igor C. A. Souto‐Santos**: Conceptualization; methodology; data curation; investigation; writing—review and editing. **Paulo A. Buckup**: Conceptualization; investigation; data curation; writing—review and editing. **Juliano Ferrer**: Conceptualization; investigation; data curation; writing—review and editing. **Vinicius J. C. Reis**: Conceptualization; investigation; writing—review and editing. **Mario de Pinna**: Conceptualization; validation; investigation; writing—review and editing; supervision. **Sergio M. Q. Lima**: Conceptualization; validation; investigation; writing—review and editing; supervision.
